# An adaptive under-frequency optimal control strategy for power system combined pumped storage and under-frequency load shedding

**DOI:** 10.1371/journal.pone.0261093

**Published:** 2021-12-09

**Authors:** Wentao Huang, Jinman Yu, Zhijun Yuan, Zhongwei He, Jun He, Minghui Deng

**Affiliations:** 1 School of Electrical and Electronic Engineering, Hubei University of Technology, Wuhan, Hubei, China; 2 Enshi Power Supply Company, State Grid Hubei Electric Power Co., Ltd., Enshi, Hubei, China; National Institute of Technology Silchar, India, INDIA

## Abstract

With the construction and development of ultra-high voltage (UHV) power grids, large-scale, long-distance power transmission has become common. A failure of the connecting line between the sending-end power grid and the receiving-end power grid will cause a large-scale power shortage and a frequency drop in the receiving-end power grid, which can result in the frequency collapse. Presently, under-frequency load shedding (UFLS) is adopted for solving the frequency control problem in emergency under-frequency conditions, which can easily cause large load losses. In this context, a frequency coordination optimal control strategy is proposed, which combines the mode transition of pumped storage units with UFLS to deal with emergency under-frequency problems. First, a mathematical model of the frequency dynamic response is established, which combines the mode transition of pumped storage units with UFLS based on a single-machine equivalent model. Then, an optimal model of the minimal area of the power system’s operation frequency trajectory is introduced, yielding the optimal frequency trajectory, and is used for obtaining the action frequency of the joint control strategy. A simulated annealing algorithm based on the perturbation analysis is proposed for solving the optimal model, and the optimal action frequency is obtained that satisfies the transient frequency offset safety constraint of the power system. Thus, the joint optimal control of the mode transition of the pumped storage units and UFLS is realized. Finally, the EPRI-36 bus system and China’s actual power grid are considered, for demonstrating the efficiency of the proposed strategy.

## 1. Introduction

The structure of China’s power grid is undergoing profound changes [1, 2], with the power grid transitioning toward ultra-high voltage (UHV) AC/DC connectivity. In 2020, China put forward the construction goals of "carbon peak" and "carbon neutralization", which are likely to accelerate the construction of the UHV power grid and the formation of a novel power system with new energy sources as the main body in the future. This change also implies structure variations, random load demands, nonlinearities, parametric ambiguities, steadily escalating size, and intricacy of the interconnected power system [3]. The randomness and volatility of new energy sources increases the demand of the grid for power-load coordination and balance. In particular, the trans-regional power transmission network transmits energy from high-capacity photovoltaic, wind, and other sources to the load center of the receiving power grid, via the UHV tie line. If the UHV tie line is locked or shut down owing to a fault, a large power shortage in the receiving power grid is likely to occur, resulting in a rapid decline in the system frequency.

At present, the main frequency modulation modes of power grids are primary frequency modulation [4] and secondary frequency modulation [5], while under-frequency load shedding (UFLS) has been the final safety control measure for preventing frequency collapses.

Among the above modes, automatic generation control (AGC) [3, 6], which takes place in secondary frequency modulation, as a macro-control means of the power grids’ large-scale coordination, has been widely studied in interconnected power systems and regional power systems, for realizing grid source coordination. However, in the current research on frequency control using AGC [7, 8], the available capacity for coordinated resources is small, and the power-grid frequency regulation in large-scale power shortage situations is limited. In [9], the kinetic energy stored in a fan impeller and a rotor shaft system was used for the primary frequency regulation of the power grid. However, owing to the limitation on the rotor speed, the provided frequency support ability is weak, and if too much of the rotor kinetic energy is released, serious stability accidents can occur. Some scholars have also adjusted the transmission power of the back-to-back interconnected system to achieve an active frequency response by means of the direct current (DC) feeding [10]. The results of these studies showed that the active frequency response control approach is mainly applicable to wide-area and/or cross-regional large-scale systems with obvious spatiotemporal distribution of the frequency, and the DC modulation effect between regions is weakened under the zero-sum game. However, in actuality it is preferred to ensure a relatively rapid frequency support and a relatively synchronous frequency response through an interconnected regional power grid with a short electrical distance. Therefore, large-capacity energy storage equipment is of great concern [11, 12]. Owing to the uncertainty of the system disturbance, after all means (such as the system reserve capacity or the system tie-line power mutual benefit) have been used for frequency control, the system may still experience a power shortage, and the system frequency may continue to decline. The power system will inevitably adopt UFLS for the frequency emergency control, thus preventing the system from exhibiting a frequency collapse.

The research on UFLS generally focuses on solving the UFLS parameters (load-shedding stages, load-shedding amount, frequency threshold, and delay) by using an optimization algorithm. In [13], a mixed integer linear programming (MILP) algorithm was used for the load-shedding optimization, according to the load priority (non-critical, semi-critical, or critical) and the load voltage stability index. In [14], the particle swarm optimization (PSO) algorithm was used for estimating the real-time unbalanced power and the load-shedding amount, and the optimal load-shedding time was calculated. In [15], a new continuous UFLS scheme was proposed, based on the traditional stage-by-stage UFLS scheme, and the nonlinear factors of the frequency threshold and time delay were considered. On this basis, an adaptive UFLS scheme was proposed in [16], based on the advanced metering infrastructure (AMI) in a smart grid, and the load-shedding process was calculated accurately in real time, to effectively reduce the control delay. The above UFLS strategies can realize emergency frequency control for preventing frequency collapses. However, these strategies are decoupled from the active frequency control means of the system after disturbance, and the coordinated control of the system frequency is not realized in combination with the above-mentioned reserve capacity. As a result, significant load shedding occurs prematurely, the control cost is high, and coordinated control with the interconnected power system is lacking.

To cope with the frequency control of power systems with large-scale power shortages and with the disadvantages of the above existing control methods, considering that the pumped storage unit features large regulating power and fast response speed, and can be converted from the pump mode to the generator mode, it has double regulation ability. In the case of an emergency fault, the ability of the pumped storage unit to suppress the initial trend of the frequency to decline by employing under-frequency pump shedding is better than that of UFLS. Therefore, it is expected that the emergency frequency control of the system can be realized through the coordination strategy of the local pumped storage unit and UFLS, which can minimize the load loss while ensuring the frequency safety of the system. References [17, 18] presented a control method for a pumped storage unit in the switching stages of the generator mode and pump mode, and the setting of control parameters of the pump-turbine governing system. In [19], the pumped storage unit was controlled for participating in the frequency control mechanism, which facilitated AGC-controlled coal-fired generators, to increase the ramping capability by switching its operation modes and generating output power variation. In [20, 21], the feasibility of a pumped storage power station to support the system frequency by changing the operation modes under severe system accidents was studied, and it was verified that the pumped storage power station could reduce the amount of load-shedding by switching the working state.

Based on the existing research on the emergency low-frequency problem of large power shortages in receiving-end power grids, this paper proposes a frequency-coordinated optimization control strategy, combining the mode conversion of pumped storage units and UFLS. First, a mathematical model of the frequency dynamic response is established, which combines the mode transition of pumped storage units with UFLS based on a single-machine equivalent model. Then, the optimal frequency control trajectory is used as the operation basis of the joint control method. A simulated annealing algorithm based on the perturbation analysis is proposed for solving the optimal model, and the optimal action frequency point is obtained under the condition of ensuring the transient frequency offset safety of the power system. Thus, the joint optimal control of the pumped-storage unit mode transition and UFLS is realized. Finally, the effectiveness of the proposed strategy is validated in simulations, which provides a frequency recovery solution for large power shortage events such as UHV receiving-end grid faults.

The main objectives and the innovative contributions of this work are as follows:

1. To recommend a frequency-coordinated optimization control strategy that combines the mode conversion of pumped storage units and UFLS for treating large power shortages.
2. The dynamic frequency variation process of the pump mode and generator mode of a pumped-storage unit is analyzed.
3. The entire process frequency dynamic response mathematical model that combines the mode conversion of pumped storage units and UFLS is established, and a control strategy based on dynamic frequency trajectory control is formulated.
4. The response characteristics and control cost of a pumped storage unit and UFLS strategy are analyzed for the grid frequency control requirements of different time scales after the fault.
5. The proposed strategy can improve the adaptability of the UFLS scheme to the power grid following large power shortages, and can help solve the emergency low-frequency problem caused by large power shortages. Compared with the traditional single UFLS control, the receiving power grid can recover the system frequency more quickly and effectively.

The remainder of this paper is organized as follows. Section 2 describes the derivation of the frequency dynamic response equation of a power system based on a single-machine model. Section 3 establishes the mathematical model of the mode transition of pumped storage units by analyzing the mode transition characteristics of the pumped storage units. Section 4 establishes the joint optimal control model, which combines pumped storage and UFLS. The joint optimal control model is solved using the simulated annealing algorithm and perturbation analysis in Section 5. Section 6 presents simulation-based verification results of the proposed strategy. The study conclusions are presented in Section 7.

## 2. Frequency dynamic response equivalent model of a power system

**Fig 1:**
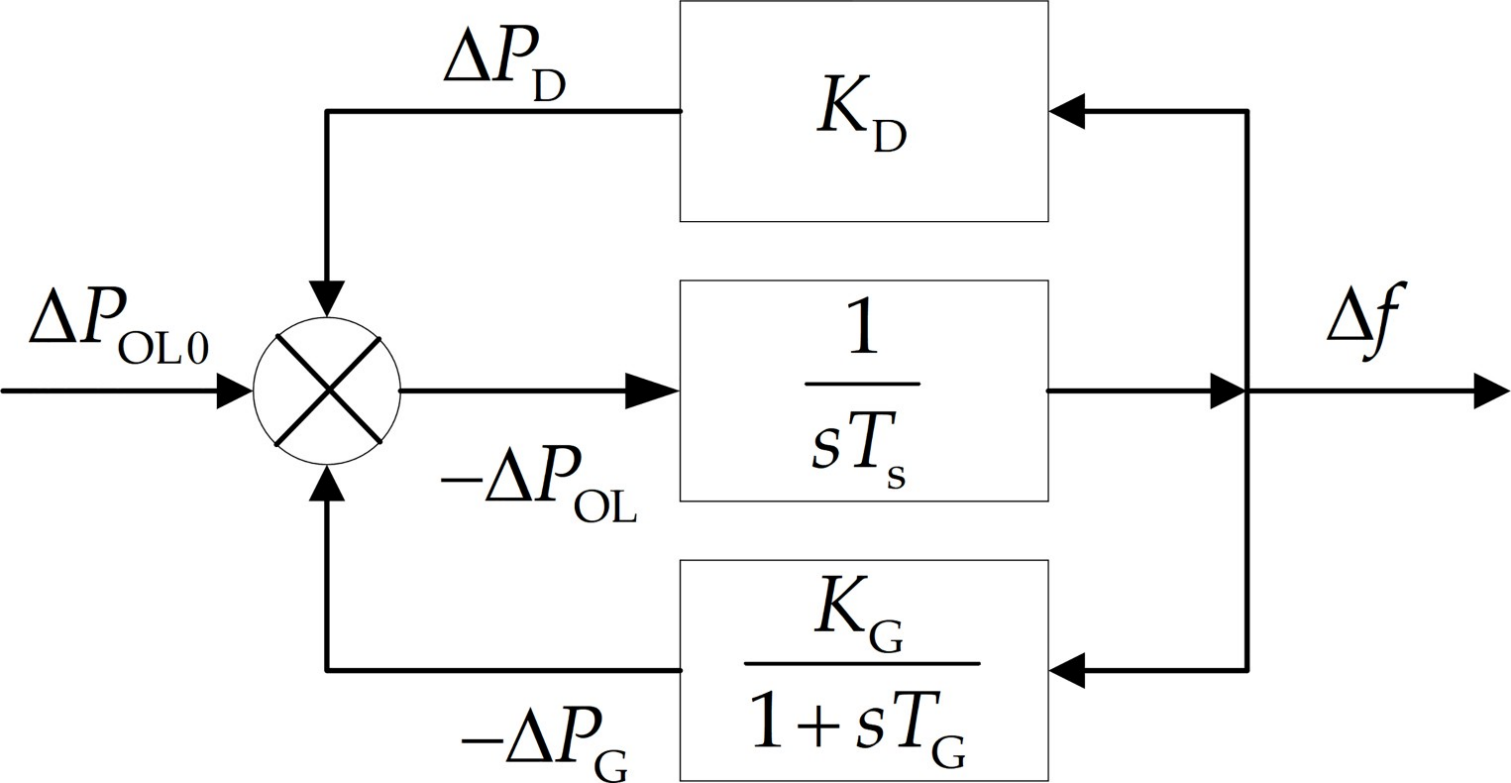
Model block diagram of the single-machine system.

The frequency of a power grid will drop when a large-scale active power shortage will occur, and an UFLS device will be activated if the frequency will continue to drop toward the threshold [22, 23]. In this study, an entire power system was considered to be equivalent to a single-machine system, for studying the frequency dynamics of the power system. The single-machine equivalent model was modeled considering the load change, the primary frequency adjustment process of the generator, and the overload of the power grid. The model is schematically shown in Fig 1.

The mathematical model of the single-machine system can be expressed by the following equations:

$$\{\begin{array}{c} T_{s}\frac{d\Delta f}{\mathrm{dt}}=-\Delta P_{\mathrm{OL}}\\ T_{G}\frac{d\Delta f}{\mathrm{dt}}+\Delta P_{G}=-K_{G}\Delta f\\ \Delta P_{D}=K_{D}\Delta f\\ \Delta P_{\mathrm{OL}}=\Delta P_{D}-\Delta P_{G}+\Delta P_{\mathrm{OL}0} \end{array}$$ (1)

where *T*_s_ is the inertial time constant of the equivalent system; Δ*f* is the frequency variation; Δ*P*_OL_ is the overload or the power shortage of the system; *T*_G_ is the comprehensive time constant of the system-wide generator set; Δ*P*_G_ is the variation in the generator power; *K*_G_ is the power-frequency static characteristic coefficient of the generator set; Δ*P*_D_ is the load variation; *K*_D_ is the load frequency coefficient; and the system’s initial overload or the initial power shortage Δ*P*_OL0_ can be expressed by the difference between the initial load *P*_D0_ and the initial generator power output *P*_G0_, Δ*P*_OL0_ = *P*_D0_−*P*_G0_.

According to Eq (1), the receiving-end grid is a typical first-order inertial system owing to the introduction of *K*_D_. A large power shortage in the receiving-end grid occurs when the UHV connecting line fails, resulting in a sharp and rapid decline in the frequency. The frequency dynamic response process can be characterized by the following full-response equation:

$$f(t)=f_{\infty }+(f_{N}-f_{\infty })e^{-\frac{1}{T_{s}}t}$$ (2)

where *f*_∞_ is the post-fault steady-state frequency of the power system; *f*_*N*_ is the rated frequency of the power system, and is also the initial frequency before the fault.

We introduce $T_{f}=\frac{T_{s}}{K_{D}}$, *K*_s_ = *K*_D_+*K*_G_, where *T*_*f*_ is the time constant during the system’s frequency decline and *K*_s_ is the comprehensive power-frequency regulation coefficient. According to Eq (1), the frequency dynamic response process curve can be obtained using Eq (3), as follows:

$$f(t)=f_{N}-\frac{\Delta P_{\mathrm{OL}0}}{K_{s}}(1-2A_{m}e^{\alpha t})\cos(\beta t+\varphi )$$ (3)

$$\{\begin{array}{c} \alpha =-\frac{1}{2}(\frac{K_{D}}{T_{s}}+\frac{1}{T_{G}})\\ \beta =\sqrt{\frac{4K_{G}T_{G}T_{s}-{\left(K_{D}T_{G}-T_{s}\right)}^{2}}{2T_{G}T_{s}}}\\ A_{m}=\frac{\sqrt{K_{G}K_{s}}}{2T_{s}\beta }\\ \varphi =a\mathrm{rctg}[\frac{1}{\beta }(\frac{1}{2}(\frac{K_{D}}{T_{s}}-\frac{1}{T_{s}})+\frac{K_{G}}{T_{s}})],\varphi \in (-\pi ,\pi ) \end{array}$$ (4)

## 3. Characteristic analysis of the operation mode transition of the pumped storage unit

### 3.1. Equivalent model of the pumped storage unit

The pumped storage unit has a large capacity, fast response, and good frequency regulation. Its dynamic behavior is closely related to the dynamic characteristics of water flow. In this study, the wave effect of the water flow was ignored, and the mechanical power loss of a hydro-turbine was considered. The following nonlinear model of a hydraulic turbine was used:

$$\left\{ \begin{array}{l} P_{\mathrm{mech}}=K_{P}\mathrm{DU}-P_{0}\\ U=K_{U}\sqrt{D}\mu_{c}\\ \frac{\mathrm{dU}}{\mathrm{dt}}=-\frac{g}{L}[D-D_{0}] \end{array}\right.$$ (5)

where ***P*_mech_** is the mechanical power output of the hydraulic turbine; *K*_P_, *K*_U_ are the proportionality coefficients; *D* is the net head of the hydraulic turbine; *D*_0_ is the steady-state initial value of, *D*; *U* is the water flow rate; *P*_0_ is the no-load loss; *μ*_*c*_ is the ideal guide vane opening; *g* is the gravity acceleration; and *L* is the length of the penstock.

The no-load loss *P*_0_ is mainly caused by the static friction force of the hydraulic turbine; as a result, there is an approximate linear relationship between the ideal guide vane opening *μ*_*c*_ and the actual guide vane opening *y*. The mathematical relationship is as follows:

$$\left\{ \begin{array}{l} P_{0}=K_{P}DU_{\mathrm{NL}}\\ U_{\mathrm{NL}}=A_{t}y_{\mathrm{NL}}\sqrt{D_{0}}\\ A_{t}=1/\left(y_{\mathrm{FL}}-y_{\mathrm{NL}}\right) \end{array}\right.$$ (6)

$$\mu_{c}=A_{t}y$$ (7)

where *U*_NL_ is the critical flow rate of the hydraulic turbine from the stationary to rotating state; *A*_*t*_ is the proportionality coefficient; *y*_FL_ is the actual full-load opening; and *y*_NL_ is the actual no-load opening of the hydraulic turbine guide vanes.

### 3.2. Operation mode transition properties of the pumped storage unit

**Fig 2:**
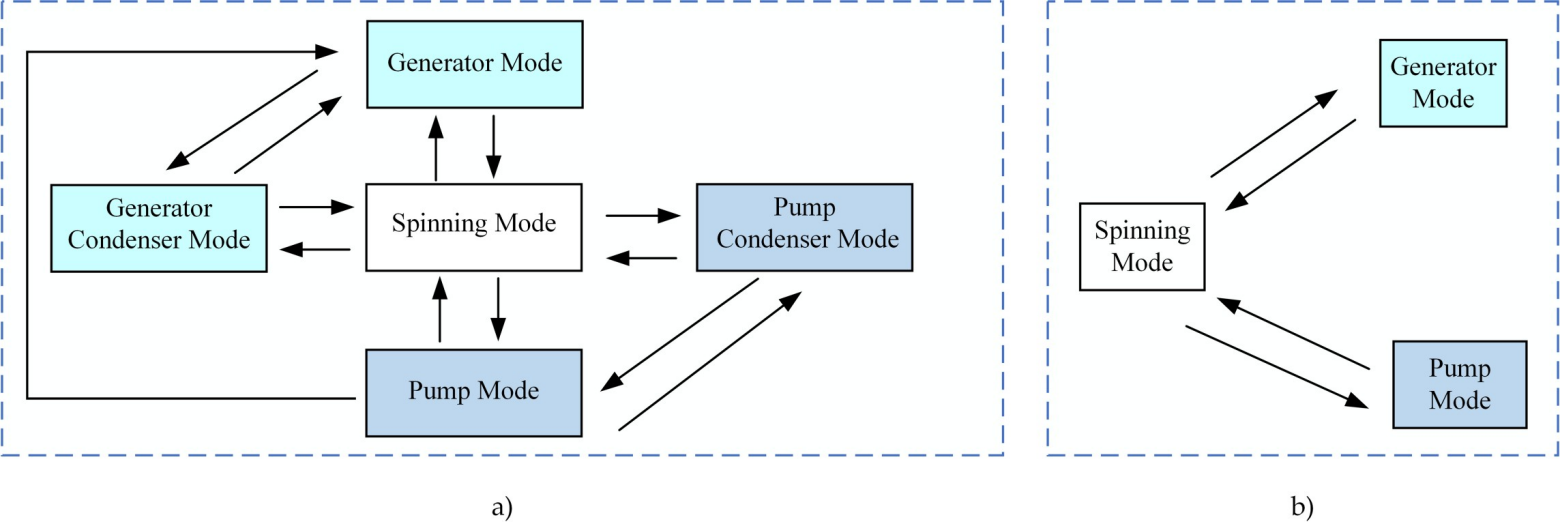
Operation mode transition diagrams of pumped storage units. (a) A typical mode transition diagram. (b) The mode transition diagram for frequency control.

As shown in Fig 2(A), the main operation modes of pumped storage units are the pump mode, the pump condenser mode, the generator mode, the generator condenser mode, and the spinning mode. Pumped storage units can contribute to the emergency frequency control by using the power output from the mode transition between the spinning, generator, and pump modes, as is schematically shown in Fig 2(B).

Suppose there are *N* pumped storage units in a power plant. The same power plant cannot have pumped storage units operating in the generator mode and the pump mode at the same time. The initial conditions are that there are *M* units in the pump mode, *N* minus *M* units in the spinning mode, and 0 units in the generator mode.

#### 3.2.1. The mode transition process from the pump mode to the spinning mode

In the steady-state pump mode of pumped storage units, the absorbed power *P*^P-S^ from the grid satisfies the power-balance Eq (8):

$$P^{P‐S}=P_{\mathrm{mech}}+P_{0}$$ (8)

There is a closing process of the guide vane mechanism when the pump mode changes to the spinning mode, and the guide vane opening *y*_P_(*t*) obeys the time-dependent linear closure law [24, 25], as follows:

$$y_{p}(t)=y_{0}-k_{y}t$$ (9)

where *y*_0_ is the initial opening and *k*_*y*_ is the proportionality coefficient of the closing process.

From Eqs (5)–(9), the time-dependent output power of the pumped storage unit changing from the pump mode to the spinning mode can be obtained using Eq (10), as follows:

$$P^{P-S}(t)=K_{p}DK_{U}\sqrt{D}A_{t}y_{p}(t)$$ (10)

Then, the *M* pumped storage units in the pump mode change to the spinning mode, stage by stage. Correspondingly, in this process, the frequency recovery process consists of *M* steps. The quantities $f_{m}^{P-S}$ and $t_{m}^{P-S}$ are the action frequency value and the action time of the *m-*th unit for transitioning from the pump mode to the spinning mode, respectively. The quantity $P_{m}^{P-S}$ is the active power contribution of the *m-*th unit to the grid, after the operation mode transformation had completed.

According to Eq (10), the power increment Δ*P*^P−S^ of the receiving-end grid after *M* pumped storage units had converted can be obtained as follows:

$$\Delta P^{P-S}=\sum_{m\mathrm{=1}}^{M}P_{m}^{P-S}$$ (11)

#### 3.2.2. The mode transition process from the spinning mode to the generator mode

According to Eq (5), the output power of a pumped storage unit is *P*^*S*−*G*^ = *P*_mech_ when in the steady-state generator mode. There is an opening process of the guide vane mechanism when the spinning mode changes to the generator mode, and the guide vane opening *y*_G_(*t*) obeys the time-dependent linear opening law, as follows:

$$y_{G}(t)=\{\begin{array}{c} k_{c}t,0\leq t<\frac{y_{\mathrm{FL}}}{k_{c}}\\ y_{\mathrm{FL}},t\geq \frac{y_{\mathrm{FL}}}{k_{c}} \end{array}$$ (12)

where *k*_*c*_ is the proportionality coefficient of the opening process.

Using Eqs (5)–(7) and Eq (12), the time-dependent output power of a pumped storage unit that transitions from the spinning mode to the generator mode can be obtained from Eq (13), as follows:

$$P^{S-G}(t)=K_{P}D[K_{U}\sqrt{D}A_{t}y_{G}(t)-U_{\mathrm{NL}}]$$ (13)

Currently, there are *N* pumped storage units in the spinning mode that transition to the generator mode, stage by stage. Correspondingly, in this process, the frequency recovery process consists of *N* steps. The quantities $f_{n}^{S-G}$ and $t_{n}^{S-G}$ are the action frequency value and the action time of the *n-*th unit for converting from the spinning mode to the generator mode, respectively. The quantity $P_{n}^{S-G}$ is the active power contribution of the *n-*th unit to grid, after its operation mode had converted.

According to Eq (13), the power increment Δ*P*^S−G^ of the receiving-end grid after *N* pumped storage units had converted can be obtained as follows:

$$\Delta P^{S-G}=\sum_{n=1}^{N}P_{n}^{S-G}$$ (14)

By substituting Eqs (11) and (14) into Eq (3), the frequency dynamic response process can be obtained for the corresponding operation mode.

## 4. Joint optimal control strategy combining the pumped storage unit and UFLS

The traditional UFLS is a passive response-based control measure that requires the system frequency to fall to a predetermined threshold before triggering the action. It is usually used for safety and stability control under extreme serious faults, with late action timing and large load shedding. This study makes full use of the mode transition properties of the pumped storage unit and UFLS, and proposes a joint optimal control strategy. When the frequency drops to a certain extent, the mode transition of the pumped storage unit is actively initiated for frequency adjustment. The UFLS is added for frequency regulation, if the frequency continues to decrease.

### 4.1. Traditional under-frequency load-shedding scheme

**Fig 3:**
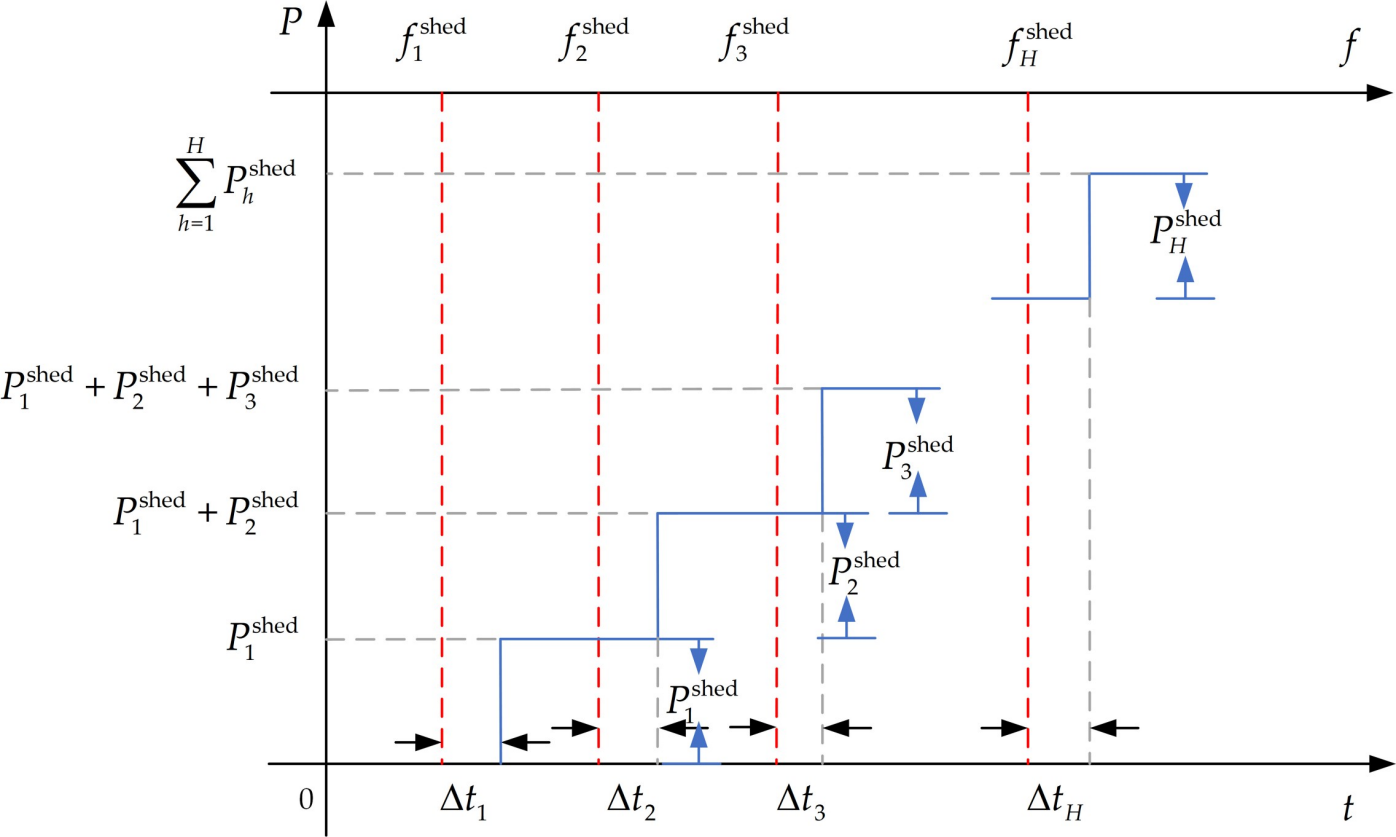
Principle of the under-frequency load shedding.

The UFLS is a traditional frequency emergency control method for power systems. It removes a certain load to reduce the active power shortage, so as to keep the frequency of the system within the non-accident range of values, and to ensure the power supply reliability of important loads [26]. It is also known as the third line of defense of the power system [27]. When a serious active power shortage occurs in the power system, the traditional UFLS scheme mainly consists of four parameters: 1) the number of stages (*H*), 2) the load-shedding value of the *h-*th stage ($P_{h}^{\mathrm{shed}}$), 3) the action frequency point of the *h-*th stage ($f_{h}^{\mathrm{shed}}$), and 4) the time delay of the *h-*th stage (Δ*t*_*h*_); this is shown in Fig 3.

### 4.2. The proposed joint optimal control strategy

**Fig 4:**
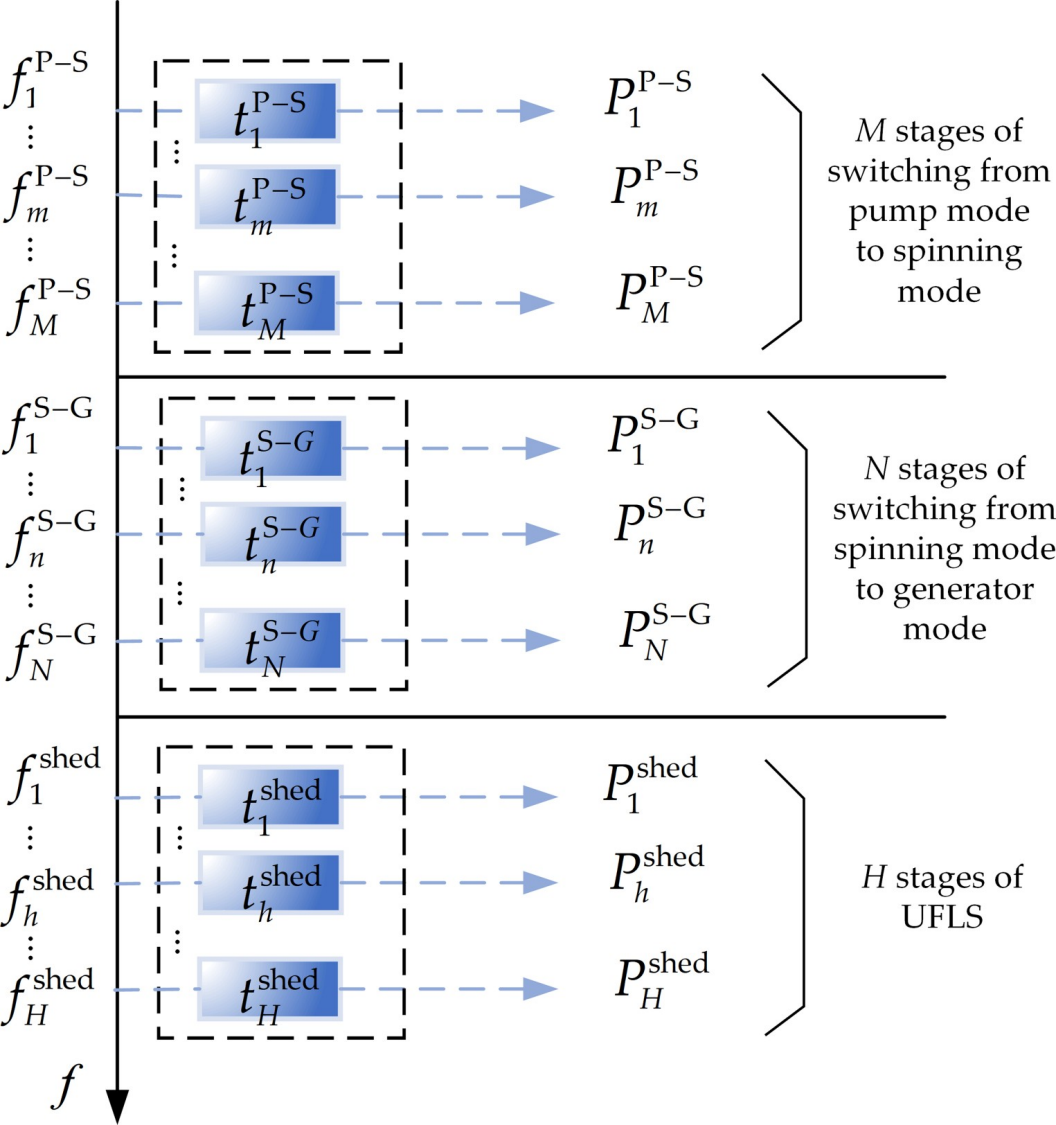
The coordination control scheme.

Consider the case of an emergency under-frequency problem in the power grid, as described in Section 2.2. First, *M* pumped storage units in the pump mode change their mode to the spinning mode, one by one. After the mode change had completed, there are *N* pumped storage units in the spinning mode. Then, if the frequency continues to decrease, the *N* units change their mode to the generator mode, one by one, for power support. However, if the frequency continues to decrease, the UFLS is triggered (consisting of *H* stages). The coordination control scheme is formulated as shown in Fig 4.

### 4.3. The realization of the joint optimal control strategy

#### 4.3.1. Dynamic frequency trajectory analysis of the joint optimal control strategy

**Fig 5:**
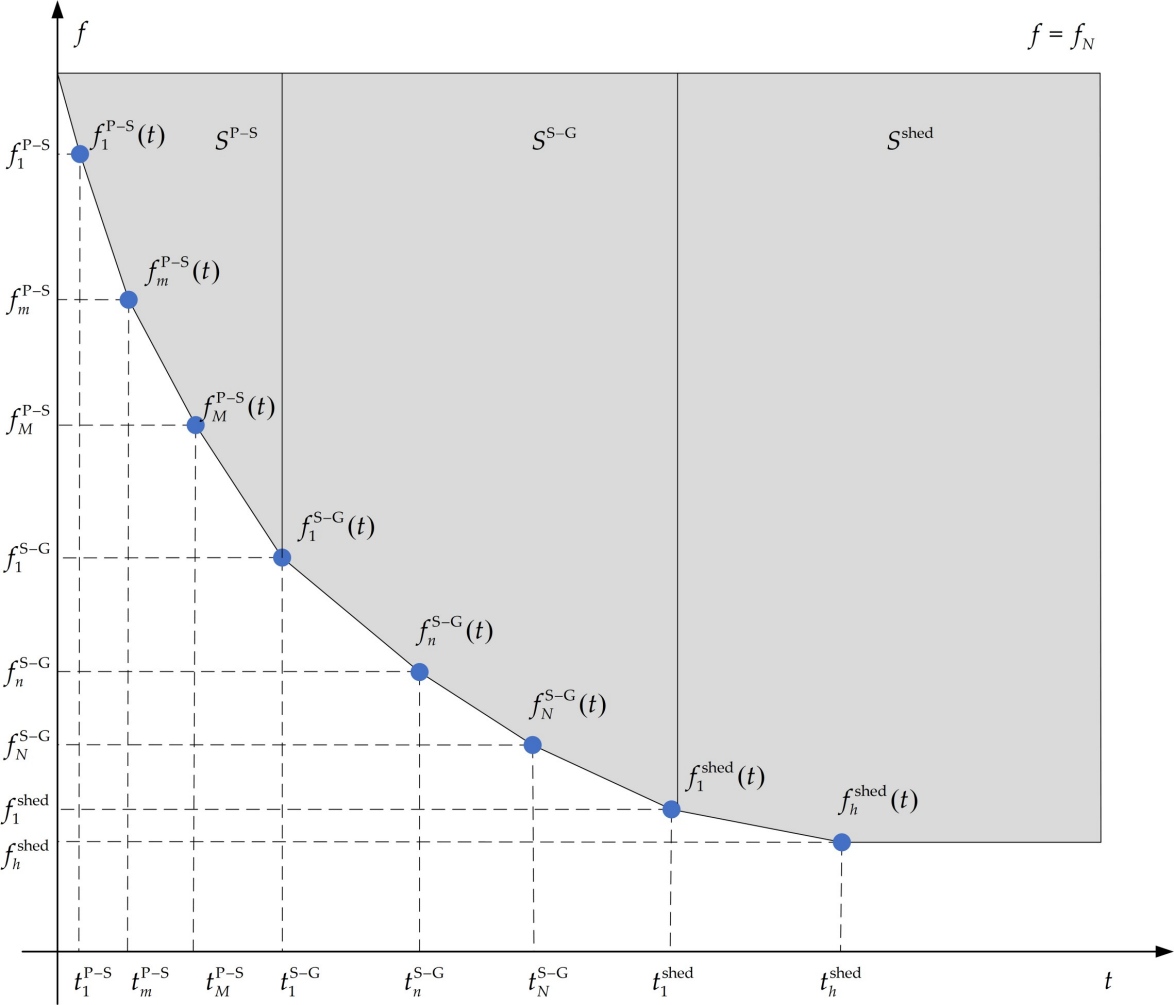
The dynamic frequency trajectory curve for the combined system of pumped storage units and UFLS.

When a failure causes a power shortage, the frequency dynamic response curve satisfies the mathematical relation in Eq (3). According to the frequency coordinated control scheme in Fig 4, the dynamic frequency trajectory curve combined with the pumped storage units with UFLS can be described as in Fig 5.

The specific analysis is as follows:

1) The initial power shortage caused by the fault is Δ*P*_OL0_, and the units change their mode from the pump mode to the spinning mode, stage by stage. The real-time power shortage of the system can be obtained from Eq (15), during the *m-*th stage of the pumped-storage unit mode transition.


$$\Delta P_{\mathrm{OL}(m)}^{P-S}=\Delta P_{\mathrm{OL}0}-\sum_{i=1}^{m}P_{i}^{P-S}$$ (15)

Substituting Eq (15) into Eq (3), the dynamic frequency trajectory in the *m-*th stage of the mode transition process can be obtained as follows:

$$f_{m}^{P-S}(t)=f_{N}-\frac{\Delta P_{\mathrm{OL}(m)}^{P-S}}{K_{s}}\cdot (1-2A_{m}e^{\alpha t})\cos(\beta t+\varphi )$$ (16)

Similarly, it can be concluded that the change in the dynamic frequency trajectory in the process of the spinning mode’s change to the generator mode can be expressed by Eq (17), as follows:

$$f_{n}^{S-G}(t)=f_{N}-\frac{\Delta P_{\mathrm{OL}(n)}^{S-G}}{K_{s}}\cdot (1-2A_{m}e^{\alpha t})\cos(\beta t+\varphi )$$ (17)

$$\Delta P_{\mathrm{OL(}n)}^{S-G}=\Delta P_{\mathrm{OL}(M)}^{S-G}-\sum_{i=1}^{n}P_{i}^{S-G}$$ (18)

where $\Delta P_{\mathrm{OL}(n)}^{S-G}$ is the real-time power shortage of the system during the *n-*th stage of the pumped-storage unit’s mode change from the spinning mode to the generator mode.

2) After the frequency regulation process of N pumped storage units had completed, in extreme cases, it will be necessary to start the UFLS stage by stage. Similarly, the dynamic frequency trajectory of the *h*-th stage of the UFLS can be obtained from Eq (19), as follows:

$$f_{h}^{\mathrm{shed}}(t)=f_{N}-\frac{\Delta P_{\mathrm{OL}(h)}^{\mathrm{shed}}}{K_{s}}\cdot (1-2A_{m}e^{\alpha t})\cos(\beta t+\varphi )$$ (19)

$$\Delta P_{\mathrm{OL}(h)}^{\mathrm{shed}}=\Delta P_{\mathrm{OL}(N)}^{S-G}-\sum_{i=1}^{h}P_{i}^{\mathrm{shed}}$$ (20)

where $\Delta P_{\mathrm{OL}(h)}^{\mathrm{shed}}$ is the real-time power shortage of the power system during the *h-*th stage of the UFLS.

#### 4.3.2. The objective function and constraint conditions of the joint optimal control strategy

The purpose of the joint control strategy is to coordinate the rapidity of the system frequency recovery, to minimize the load loss, and to obtain the optimal frequency control trajectory. This goal can be achieved by reasonably determining the action frequency points in each stage. As shown in Fig 5, based on the dynamic frequency trajectory, this study defines the minimal area surrounded by the frequency trajectory curve and the rated frequency curve as the objective function. By solving the objective function, the action frequency points of each stage can be determined, and finally, the optimal frequency trajectory of the system operation can be determined point by point.

As shown in Fig 5, the gray parts of the area enclosed by the dynamic frequency trajectory and the rated frequency *f* = *f*_*N*_ are, *S*^P−S^,*S*^S−G^,*S*^shed^ respectively, representing the three stages: 1) the pump mode conversion to the spinning mode, 2) the spinning mode conversion to the generator mode, and 3) the UFLS.

Matrix ***X*** is defined, and it consists of action frequency points ***f*** and corresponding action time series ***t***, as follows:

$$\begin{array}{l} \;X=\left[\begin{array}{l} f\\ t \end{array}\right]\\ \left\{ \begin{array}{l} f=[f_{1}^{P-S},\cdots ,f_{m}^{P-S},\cdots ,f_{M}^{P-S},f_{1}^{S-G},\cdots f_{n}^{S-G},\cdots ,f_{N}^{S-G},f_{1}^{\mathrm{shed}},\cdots ,f_{h}^{\mathrm{shed}},\cdots ,f_{h}^{\mathrm{shed}}]\\ t=[t_{1}^{P-S},\cdots ,t_{m}^{P-S},\cdots ,t_{M}^{P-S},t_{1}^{S-G},\cdots t_{n}^{S-G},\cdots ,t_{N}^{S-G},t_{1}^{\mathrm{shed}},\cdots ,t_{h}^{\mathrm{shed}},\cdots ,t_{h}^{\mathrm{shed}}] \end{array}\right. \end{array}$$ (21)

The mathematical model of the objective function of the joint control strategy is established as follows:

$$\left\{ \begin{array}{l} \min\;S=S^{P-S}+S^{S-G}+S^{\mathrm{shed}}\\ s.t.\;S^{P-S}=\sum_{m=1}^{M}\int_{t_{m}^{P-S}}^{t_{m+1}^{P-S}}(f_{N}-f_{m}^{P-S}(t))dt\\ \;S^{S-G}=\sum_{n=1}^{N}\int_{t_{n}^{S-G}}^{t_{n+1}^{S-G}}(f_{N}-f_{n}^{S-G}(t))dt\\ \;S^{\mathrm{shed}}=\sum_{h=1}^{H}\int_{t_{h}^{\mathrm{shed}}}^{t_{h+1}^{\mathrm{shed}}}(f_{N}-f_{h}^{\mathrm{shed}}(t))dt\\ \;f(t)=f_{N}-\frac{\Delta P_{\mathrm{OL}}}{K_{s}}(1-2A_{m}e^{\alpha t})\cos(\beta t+\varphi ),t\in t,f(t)\in f\\ \;f_{\min}^{P-S}\leq f_{m}^{P-S}\leq f_{\max}^{P-S},f_{\min}^{S-G}\leq f_{n}^{S-G}\leq f_{\max}^{S-G},f_{\min}^{\mathrm{shed}}\leq f_{h}^{\mathrm{shed}}\leq f_{\max}^{\mathrm{shed}} \end{array}\right.$$ (22)

where $f_{\min}^{P-S},f_{\max}^{P-S},f_{\min}^{S-G},f_{\max}^{S-G}$ is the transient frequency safety limit of the action frequency of pumped storage units during the mode change and $f_{\min}^{\mathrm{shed}},f_{\max}^{\mathrm{shed}}$ are the transient frequency safety limits for the UFLS.

## 5. Solution of the joint optimal control strategy using the simulated annealing algorithm with perturbation analysis

Because the power system is a nonlinear and non-autonomous system, the calculation scale of the response problem is large after a large disturbance, which involves solving the time-domain trajectory and calculating the margin index. The traditional analytical method cannot easily deal with this problem; thus, time-domain trajectories are computed using numerical simulations, considering the time-domain trajectory constraints. According to the mathematical description of Eq (22), the optimal solution of the joint optimal control strategy problem seeks the optimal solution in the space of feasible solutions constrained by the transient frequency security. Owing to the nonlinearity and the characteristics of the optimal solution of the joint optimal control strategy, the optimization should be performed on the boundary of the space of feasible solutions, which can be used for designing the solution algorithm.

To avoid the problem of solution trapping in a local minimum and high computational complexity for large-scale solutions, the current study utilized the simulated annealing algorithm based on perturbation analysis. According to a small change in the system’s parameters, the extent of the change of the time-domain frequency trajectory is used for guiding the solution to the problem. Then, the mathematical model of the emergency frequency coordination control is transformed into a local linear programming problem, and the simulated annealing algorithm is used for solving the problem efficiently. The algorithm utilizes the properties of linear programming in boundary optimization, for obtaining an optimal solution that satisfies the imposed constraints.

The method can be used for “online budget and implement matching” control measure optimization. That is, real-time updating of the grid operation state and solving the model for obtaining the frequency control plan are done first, and then the action frequency points of each stage are distributed using a high-speed communication system. When faults are detected that meet the prescribed conditions, the plan is immediately started for maintaining the system’s security and stability.

### 5.1. Processing of constraints based on perturbation analysis

The objective function of the joint control strategy is the linear superposition of the area enclosed by each stage’s frequency trajectory and the rated frequency curve, which is a nonlinear condition satisfying the transient frequency constraint. In the process of calculating the action frequency point based on the frequency trajectory, the sensitivity [28] of the frequency trajectory that underlies time-domain variations of the trajectory is introduced based on perturbation analysis, and the transient frequency safety constraint is locally linearized. Then, the mathematical model of the joint control strategy can be transformed into a linear programming problem, for solving.

The trajectory sensitivity matrix ***A*** is defined using the perturbation analysis method, and locally linearizes the transient frequency safety constraints into the following form:

$$\eta_{f}+A\Delta f_{V}\geq \varepsilon_{f}$$ (23)

$$\left\{ \begin{array}{l} A=[a_{f1},\ldots ,a_{\mathrm{fi}},\ldots ,a_{fN_{L}}]\\ \eta_{f}=[\eta_{f1},\ldots ,\eta_{\mathrm{fi}},\ldots ,\eta_{fN_{L}}]\\ \Delta f_{V}=[\Delta f_{f2},\ldots ,\Delta f_{\mathrm{fi}+1},\ldots ,\Delta f_{fN_{L}-1},0] \end{array}\right.$$ (24)

where ***η***_*f*_ is the transient frequency safety margin vector, *η*_*fi*_ is the transient frequency safety margin when the power shortage is, Δ*P*_*i*_; Δ***f***_V_ is the frequency variation vector, Δ***f***_*i*+1_ is the frequency variation of the *i+*1th stage; ***ε***_*f*_ and is the transient frequency safety threshold value, that is, the transient frequency safety limits of Eq (22).

Among them,

$$\begin{array}{l} a_{fi}=\frac{\eta_{fi}(\Delta P_{i},\tau_{i})-\eta_{fi}(\Delta P_{i})}{\tau_{i}}\\ \eta_{\mathrm{fi}}=\min(\frac{\int_{t_{i}}^{t_{i}+t_{\mathrm{cr,i}}}(f_{i}-f_{\mathrm{cr,i}})dt}{(f_{N}-f_{\mathrm{cr,i}})t_{\mathrm{cr,i}}}) \end{array}$$ (25)

where *a*_*fi*_ represents the trajectory sensitivities of *η*_*fi*_; *η*_*fi*_(Δ*P*_*i*_) is the transient frequency deviation safety margin for the power shortage Δ*P*_*i*_ of the *i-*th stage and *η*_*fi*_(Δ*P*_*i*_,*τ*_*i*_) is the margin index *η*_*fi*_(Δ*P*_*i*_) with the power shortage Δ*P*_*i*_ of the *i-*th stage increased by *τ*_*i*_; *f*_*i*_ is the action frequency of the *i-*th stage and *f*_*cr*,*i*_ is the frequency deviation threshold of *f*_*i*_; *t*_*cr*,*i*_ is the maximal time at which the action frequency *f*_*i*_ exceeds the threshold *f*_*cr*,*i*_; and *t*_*i*_ is the action starting time of the *i-*th stage.

The computation that uses the perturbation method to obtain the trajectory sensitivity matrix ***A*** is mainly concentrated on the dynamic simulation of the perturbation of each stage’s power shortage. Because these dynamic simulations are independent of each other, the computation can be parallelized, for speeding up the solution of the trajectory sensitivity matrix.

Owing to the nonlinear nature of the problem, it is impossible to solve it once to obtain the optimal solution; yet, the boundary of the feasible solution space can be quickly reached using an optimization algorithm, allowing to find an approximately optimal solution. Based on the previous solution, the local linearization and linear programming solution determination steps were carried out again, to further approximate the optimal solution. Through repeated iterative operations and until the solution converged, the obtained solution constituted the optimal solution of the problem.

### 5.2. Simulated annealing algorithm

The simulated annealing algorithm can deal with highly nonlinear optimization problems, such as global optimization and discrete-variable optimization. It mainly requires three functions to control the iterative process: cooling time, random solution, and the Metropolis criterion [29, 30].

1. The cooling function is defined as
$$T=T_{0}*K$$ (26)
  where *T*_0_ is the initial temperature and *K* is the cooling factor.
2. The random solution ***X***, defined by Eq (21), is generated, and the frequency constraint of Eq (22) is satisfied.
3. The unsatisfactory solution is accepted by the Metropolis criterion with a certain probability.

$$prob=\{\begin{array}{l} 1\;,\Delta E<0\\ e^{-\frac{\Delta E}{T}},\Delta E>0 \end{array}$$ (27)

where *prob* is the probability that a new solution will be accepted, Δ*E* is the variation of the system energy, and $\Delta E=\Delta E^{T_{i}}-\Delta E^{T_{i}{}_{-1}}$.

### 5.3. Solution based on the perturbation analysis—simulated annealing algorithm

1. Initialize the initial temperature *T*_0_, the cooling stop temperature *T*_end_, the cooling factor *K*, the Markov chain or the iteration length *L*, and the iteration number *i* = 0.
2. At the current temperature *T*_*i*_ = *T*_0_, randomly select the initial positions ***X***_*i*_ = ***X***_0_ of those particles that satisfy the frequency constraint. Set the power shortage to Δ*P*_*i*_; set the current objective function value that can be solved, that is, the area value enclosed by the frequency trajectory and the rated frequency $S_{i}^{T_{i}}=S_{0}^{T_{0}}$.
3. Generate new particles *X*_*i*+1_ by perturbation analysis in the near subset of the current solution *X*_*i*_ and obtain the new objective function value $S_{i+1}^{T_{i}}$.
4. Calculate $\Delta E=S_{i+1}^{T_{i}}-S_{i}^{T_{i}}$. If Δ*E*<0, the action frequency value represented by the new position state is regarded as the current optimal position state. If Δ*E*>0, the new position state is accepted as the current optimal action frequency value according to the probability rule in Eq (27).
5. Search for all solutions at this temperature *T*_*i*_ and determine whether the loop end condition is satisfied. If yes, output the optimal action frequency value; otherwise, drop the temperature to *T*_*i*+1_ = *T*_i_*K; set *i* = *i*+1, set the power shortage to Δ*P*_*i*_, and repeat steps 3) to 5).

**Fig 6:**
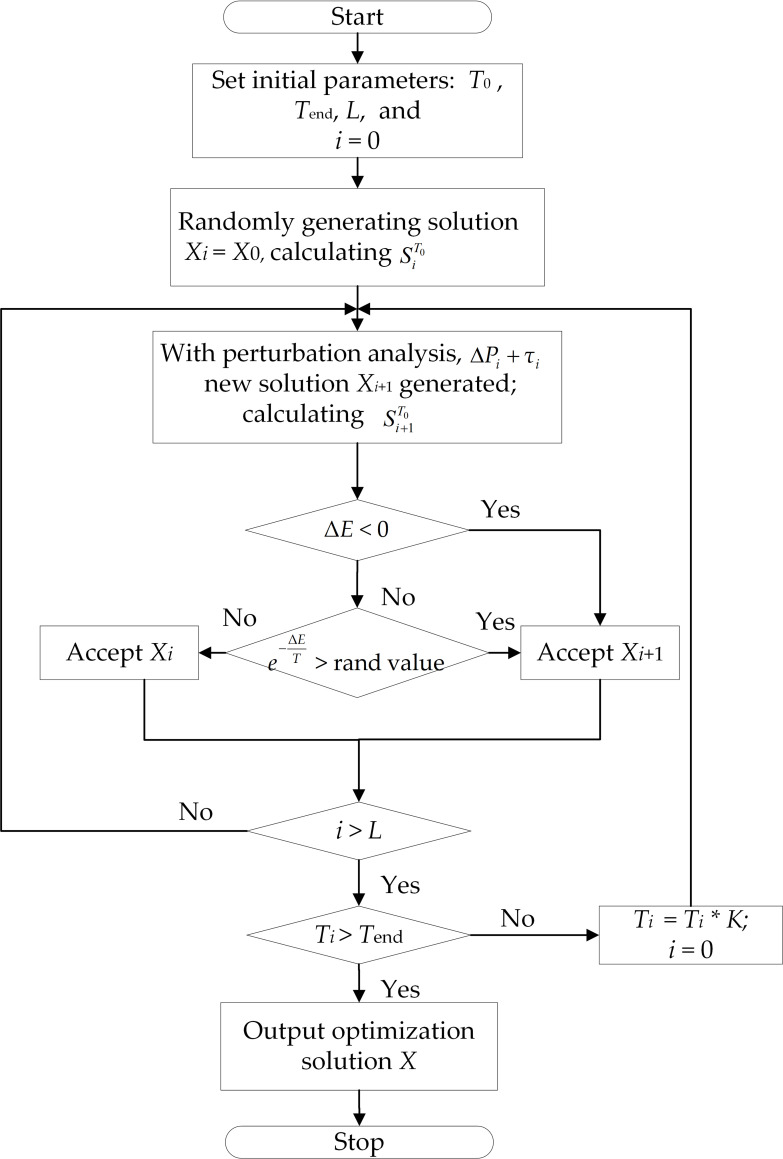
Flowchart of the solution process.

Fig 6 shows the flowchart of the solution process.

## 6. Case studies

### 6.1. Testing on the EPRI-36 bus system

**Fig 7:**
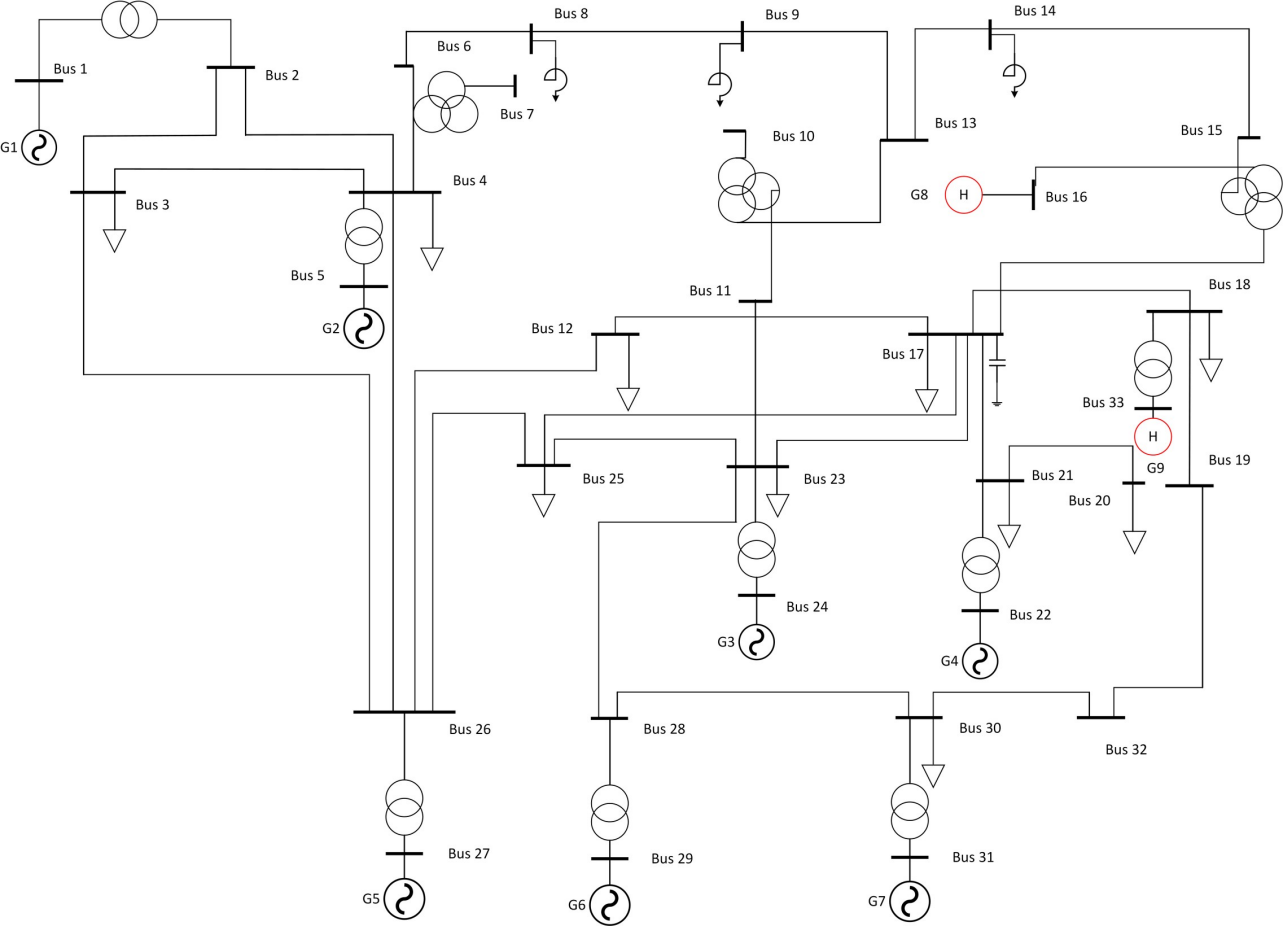
Topology of the EPRI-36 bus system.

The modified EPRI-36 bus system shown in Fig 7 was taken as an example. There were nine generators, of which G8 and G9 were pumped storage units. The initial operation modes and power outputs of the pumped storage units are listed in Table 1. The system parameters are as follows: the rated frequency *f*_*N*_ = 50Hz; inertial time constant *T*_S*_ = 7.18; the comprehensive time constant of the system-wide generator set *T*_G*_ = 3.54; the load frequency coefficient *K*_D*_ = 1.7; the power-frequency static characteristic coefficient of the generator set *K*_G*_ = 22.52.

**Table 1:** Initial parameters of the pumped storage units in the EPRI-36 bus system.

NO. of Units	Initial Mode	Initial Power Output (MW)	Power Output Range (MW)
G8	Spinning mode	0	-200~200
G9	Pump mode	-300	-300~300

**Fig 8:**
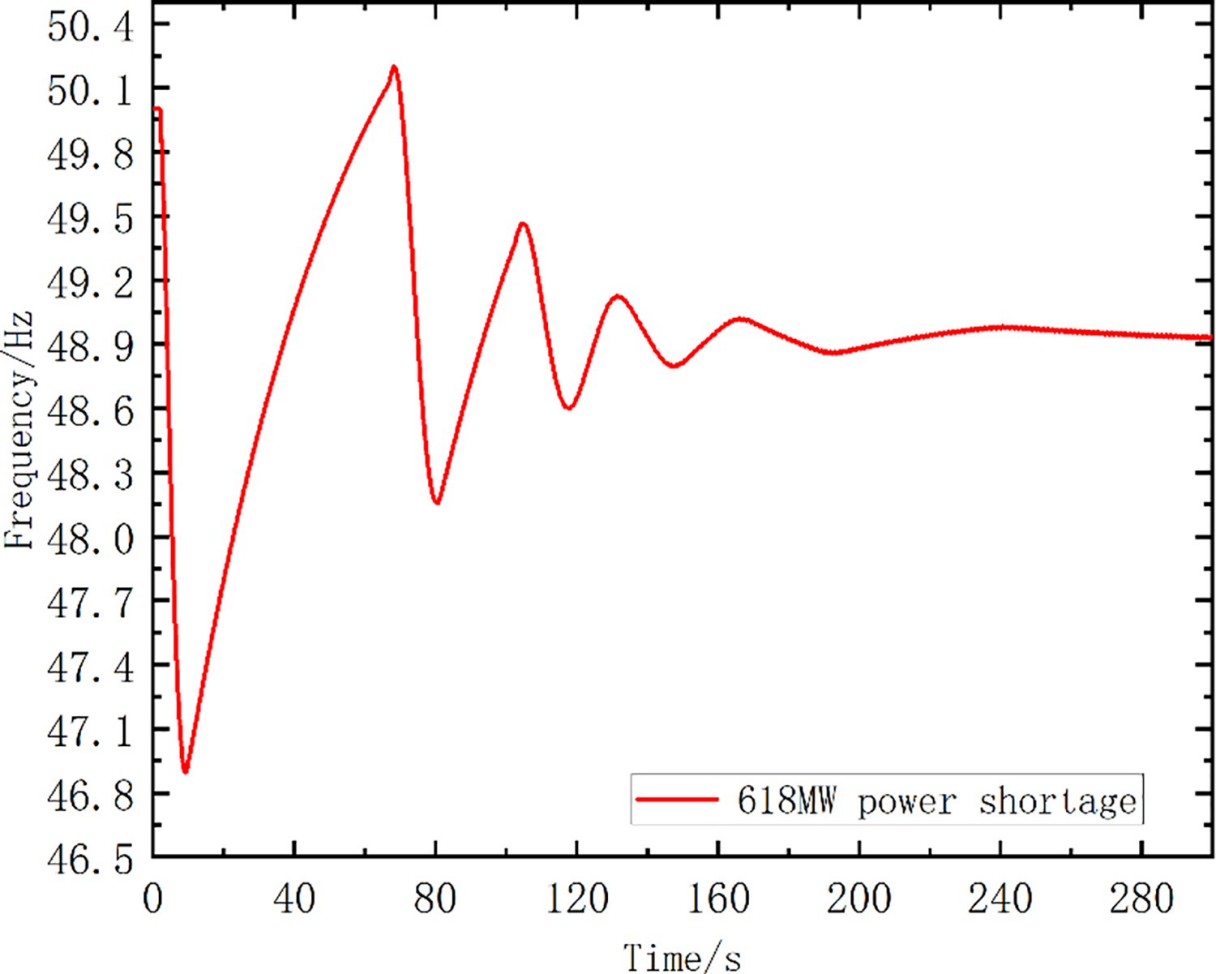
The frequency curve without any measures.

The disturbance was set as follows: the G2 unit was shut down at 2 s, resulting in a power shortage of 618 MW. Bus 11 was selected as the system frequency observation object. Fig 8 shows the frequency curve without frequency control measures.

According to the current regulation of the power grid dispatching, the rated frequency deviation of the power grid cannot exceed ±0.2 Hz, and frequencies above this range are marked as accident-related. Usually, the action value at the first stage of UFLS is 49.0 Hz, and there is no uniform regulation for the action frequency value of the last stage. Therefore, the maximal action frequency of the pumped storage units was set to 49.8 Hz, while the minimal action frequency was 49.0 Hz. Then, the maximal action frequency of the UFLS was $f_{\max}^{shed}=49.0\mathrm{Hz}$, while the minimal action frequency of the UFLS was $f_{\min}^{shed}=48.2\mathrm{Hz}$.

**Fig 9:**
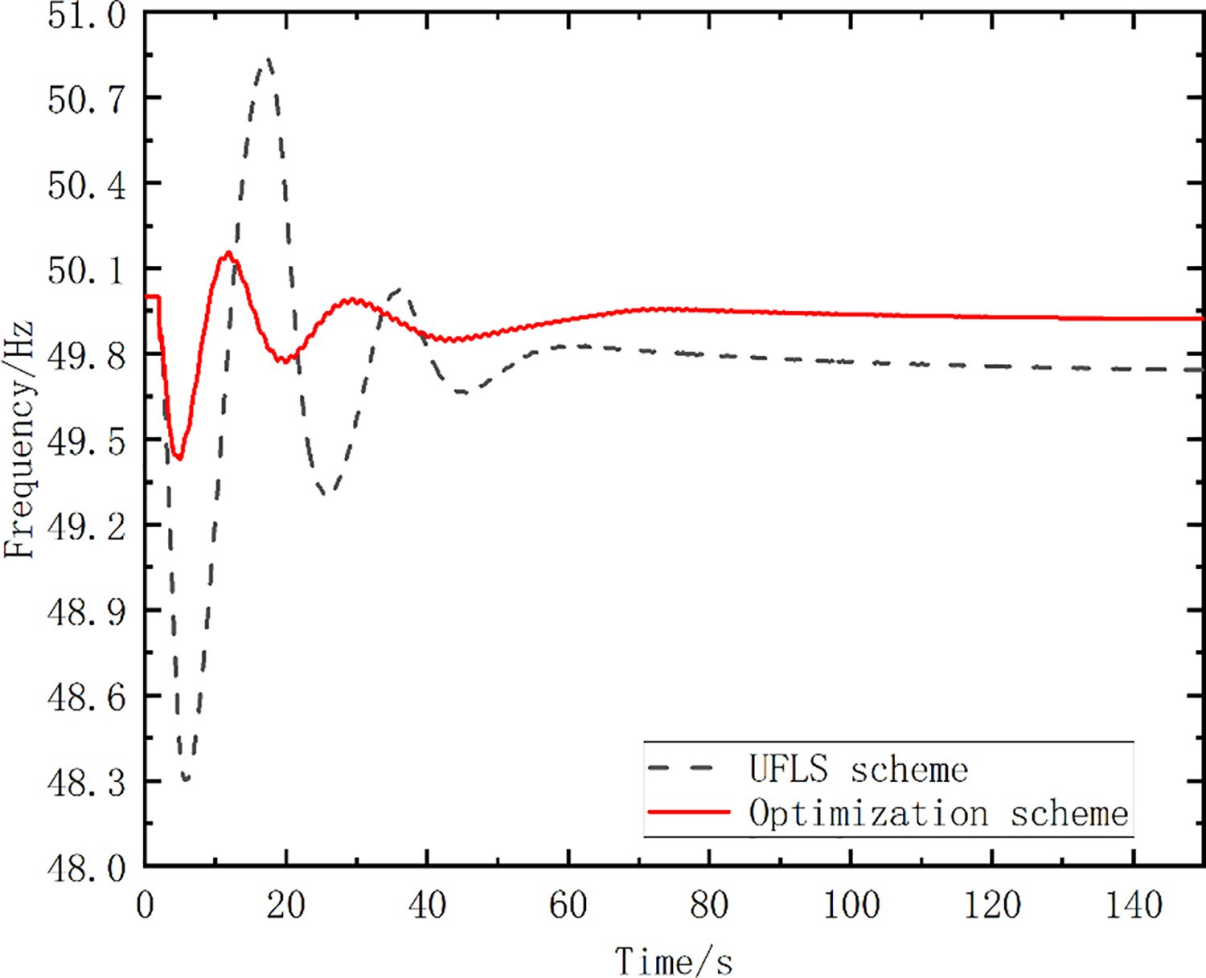
Simulated frequency curves.

The pumped storage units were divided into three stages, to participate in the frequency control using the strategy proposed in this paper. The above parameters of the system were introduced into the proposed optimal model and solved with the proposed strategy and algorithm, and one optimized scheme was obtained, as shown in Table 2, which included a group of optimal action frequency values and the number of action stages. In Table 2, the optimized scheme proposed in this paper is compared with the traditional UFLS scheme, and the action frequency setting values of the two schemes are shown. The simulated frequency curve is shown in Fig 9.

**Table 2:** Two comparison schemes.

stages	Action Frequency Setting Value (Hz)	
	Optimized Scheme	UFLS Scheme
1	49.76	49.0
2	49.74	48.8
3	49.63	48.6
4	-	48.4
5	-	48.2

To compare and analyze each scheme, several variables are defined: *f*_min_ is the minimal frequency of the frequency curve; Δ*t_re_* is the time at which the frequency returns to the steady state; *S* is the area enclosed by the entire frequency trajectory curve with *f* = *f*_*N*_. Table 3 lists the simulation analysis results.

**Table 3:** Simulation analysis results.

Schemes	*f*_min_ (Hz)	*f*_∞_ (Hz)	Δ*t_re_*(s)	*S*	Load shedding value (MW)
UFLS	48.30	49.74	131.62	36.57	494.4
Optimized Scheme	49.43	49.92	116.96	8.68	0

As shown in Fig 9 and Table 3, the lowest frequency value *f*_min_ at the first pendulum, the steady-state frequency *f*_∞_, and the time at which the frequency returns to the steady state are improved, compared with the traditional UFLS scheme. In addition, the optimized scheme only needs to perform the mode conversion of the pumped storage units without shedding the load, while the UFLS needs to shed the load of 494.4 MW.

**Fig 10:**
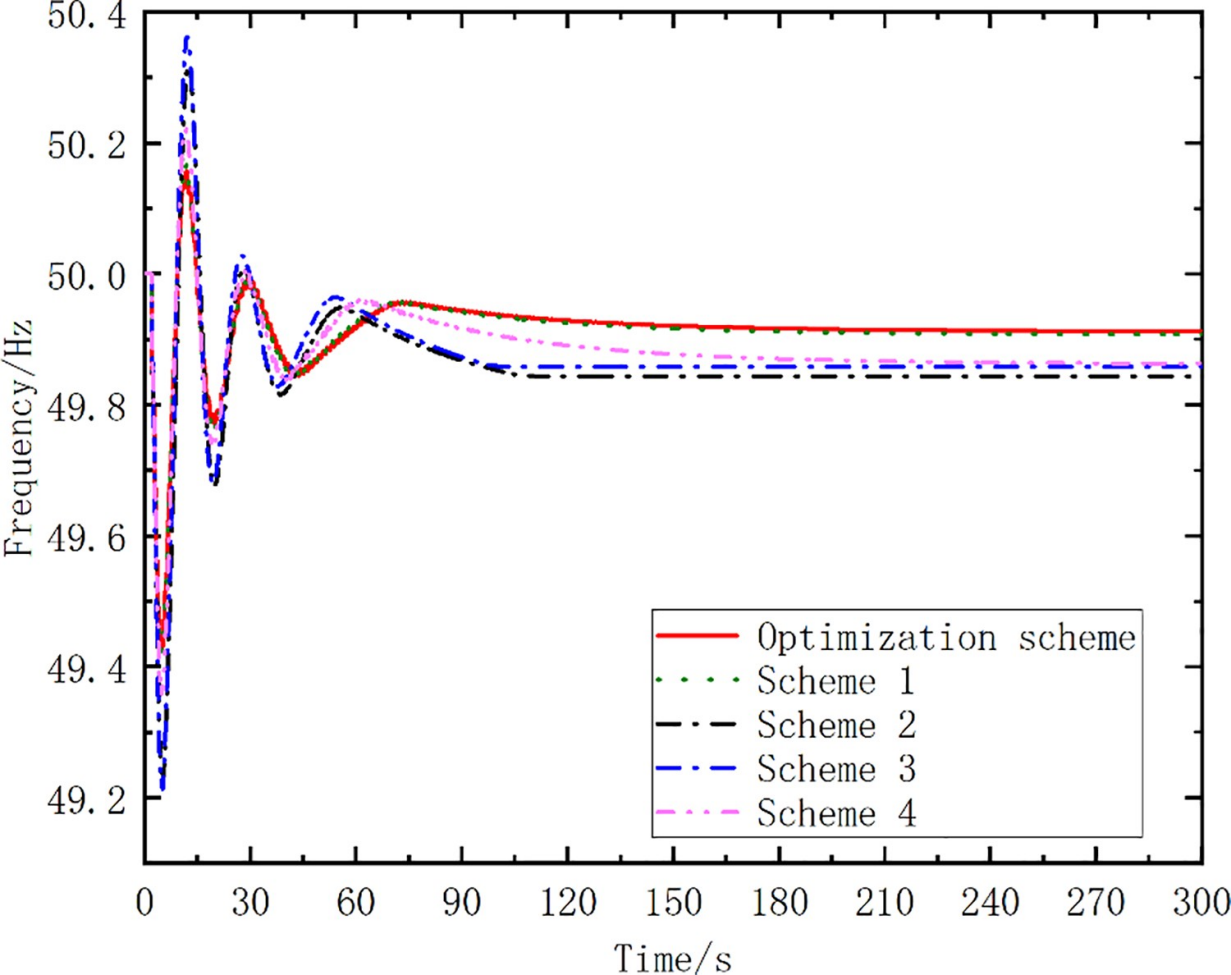
Simulated frequency curves for different schemes.

To highlight the effectiveness of the proposed optimization algorithm, the results for five schemes are compared in Table 4. The frequency action points of the pumped storage units in schemes 1 to 3 vary by 0.1, 0.2, and 0.3 Hz, respectively. The mode conversion start time exhibits a 0.1-s-long delay and varies by 0.2 Hz for Scheme 4. The last scheme is the optimized scheme proposed in this study. Fig 10 shows the simulated frequency curves for the different schemes. Table 5 lists the simulation analysis results.

**Table 4:** Comparison of five different schemes.

Stages	Action Frequency Setting Value under Various Schemes (Hz)				
	Scheme 1	Scheme 2	Scheme 3	Scheme 4	Optimized Scheme
1	49.80	49.80	49.80	49.70	49.76
2	49.50	49.70	49.60	49.50	49.74
3	49.20	49.60	49.40	49.30	49.63

**Table 5:** Simulation analysis results.

Schemes	*f*_min_(Hz)	*f*_∞_ (Hz)	Δ*t_re_*(s)	*S*
Scheme 1	49.35	49.87	143.05	19.93
Scheme 2	48.41	49.91	134.56	13.56
Scheme 3	48.21	49.84	117.12	16.10
Scheme 4	48.21	49.85	120.19	19.85
Optimized Scheme	49.43	49.92	116.96	8.68

From Fig 10 and Table 5, it can be seen that the amplitude of the first pendulum drop of the optimized scheme proposed in this paper is smaller than that of Schemes 1–4. The time of the frequency recovery to 49.92 Hz for the optimized scheme is 116.96 s, which is shorter than those for the other schemes. The curve area enclosed by the optimized scheme is the smallest for the proposed scheme. The simulation results show that the strategy proposed in this study yields good results in terms of the frequency control.

### 6.2. Tests on China’s actual power grid

**Fig 11:**
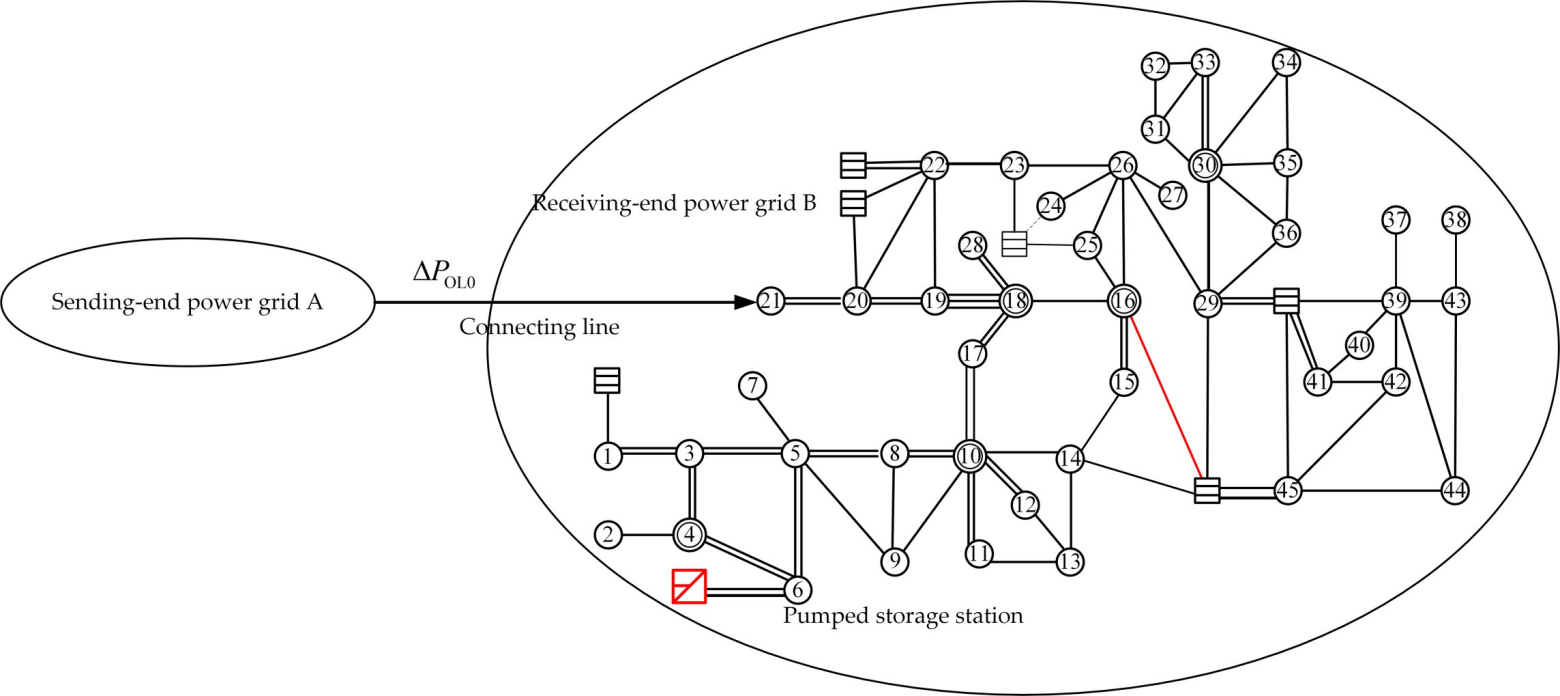
Simulated test power grid.

China’s actual power grid was used for validating the effectiveness of the joint optimal control strategy proposed for frequency recovery. As shown in Fig 11, the simulated test power grid consisted of the sending-end power grid A and the receiving-end power grid B. The substations were numbered separately, and the red line was the key simulation analysis object after the connecting line between grids A and B was made to malfunction. Power was transmitted by the AC connecting line between grid A and grid B, and the power value was Δ*P*_OL0_.

The system parameters were as follows: the rated frequency was *f*_*N*_ = 50Hz; the inertial time constant was *T*_S*_ = 8.01; the comprehensive time constant of the system-wide generator was set to *T*_G*_ = 5.21; the load frequency coefficient was *K*_D*_ = 1.9; the power-frequency static characteristic coefficient of the generator was set to *K*_G*_ = 21.6.

There were three pumped storage units in the simulated test power grid. The initial modes of the units and their power outputs are listed in Table 6.

**Table 6:** Initial parameters of pumped storage units.

NO. of Units	Initial Mode	Initial Power Output (MW)	Power Output Range (MW)
1	Pump mode	-300	-300~300
2	Pump mode	-300	-300~300
3	Spinning mode	0	-300~300

According to the control strategy established in Fig 4 and combined with the state parameters of the pumped storage units in Table 6, the pumped storage units participating in the frequency regulation of the system during the mode transition process could be divided into five stages. The UFLS was carried out by five stages of proportional load shedding according to 20% of the power shortage $\Delta P_{\mathrm{OL}}^{S-G}$ after the mode transition process had completed, which was equal to the initial power shortage Δ*P*_OL0_ minus the power contribution of the mode transition process. The stage-by-stage joint control schemes are presented in Table 7.

**Table 7:** Stage-by-stage joint control scheme.

*i*th Stage	Frequency adjustment measures	Power Contribution (MW)
1	Pump mode to spinning mode	300
2	Pump mode to spinning mode	300
3	Spinning mode to generator mode	300
4	Spinning mode to generator mode	300
5	Spinning mode to generator mode	300
6	Load shedding	20%* $\Delta P_{\mathrm{OL}}^{S-G}$
7	Load shedding	20%* $\Delta P_{\mathrm{OL}}^{S-G}$
8	Load shedding	20%* $\Delta P_{\mathrm{OL}}^{S-G}$
9	Load shedding	20%*$\Delta P_{\mathrm{OL}}^{S-G}$
10	Load shedding	20%*$\Delta P_{\mathrm{OL}}^{S-G}$

Table 7 shows the theoretical maximal number of frequency adjustment stages of the verified power system according to the proposed joint control strategy. The number of the actual frequency adjustment stages could be reduced according to the frequency recovery situation.

In the following, two scenarios of different power shortages were simulated using the power system analysis software package (PSASP), for validating and analyzing the effectiveness of the strategy proposed for the power grid frequency adjustment. The frequency of substation bus 16 in Fig 11 was taken as the observation object of the system frequency.

Case 1: Initial power shortage ΔP_OL0_ = 4000MW of receiving-end grid B

**Fig 12:**
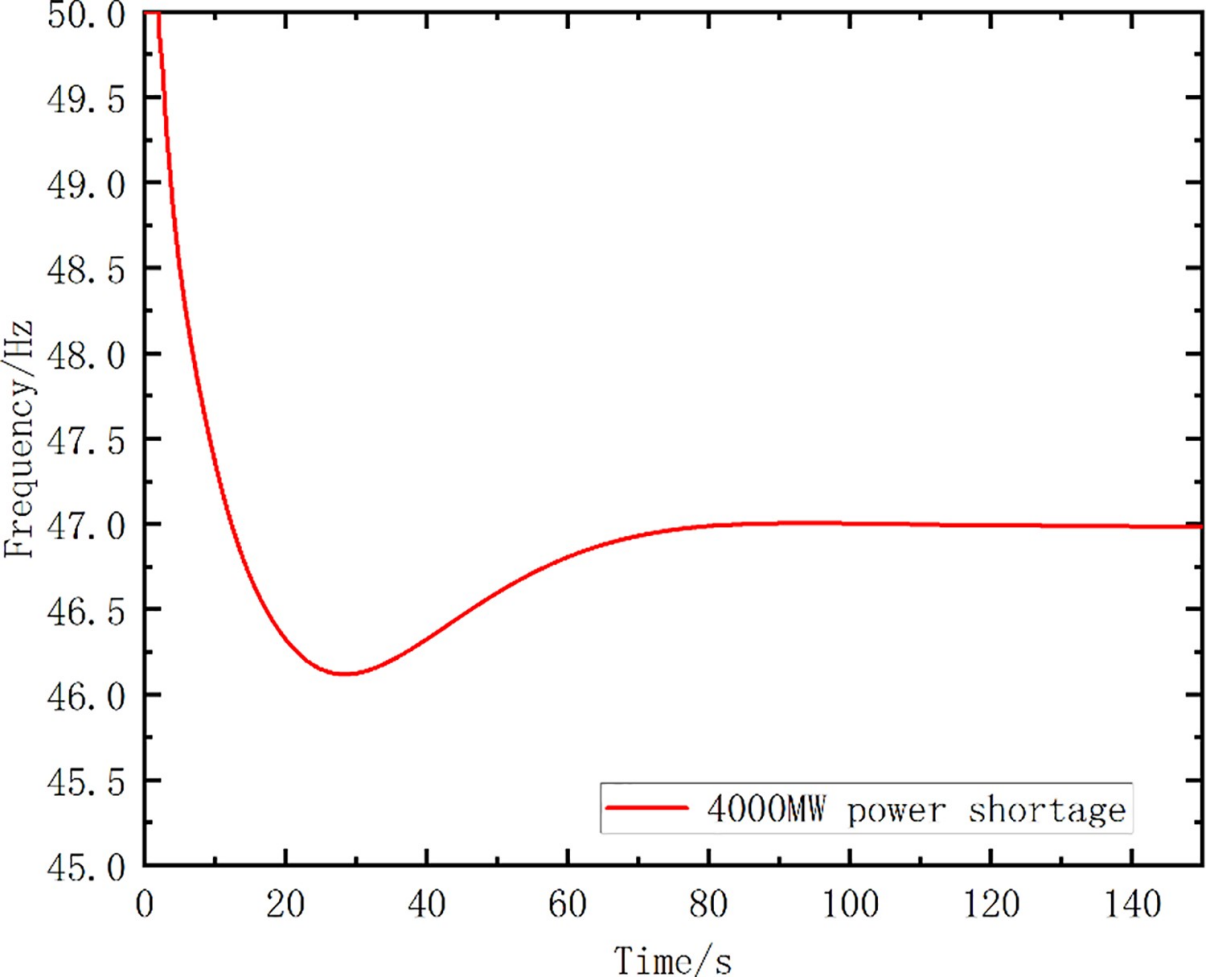
The frequency curve without any measures.

**Fig 13:**
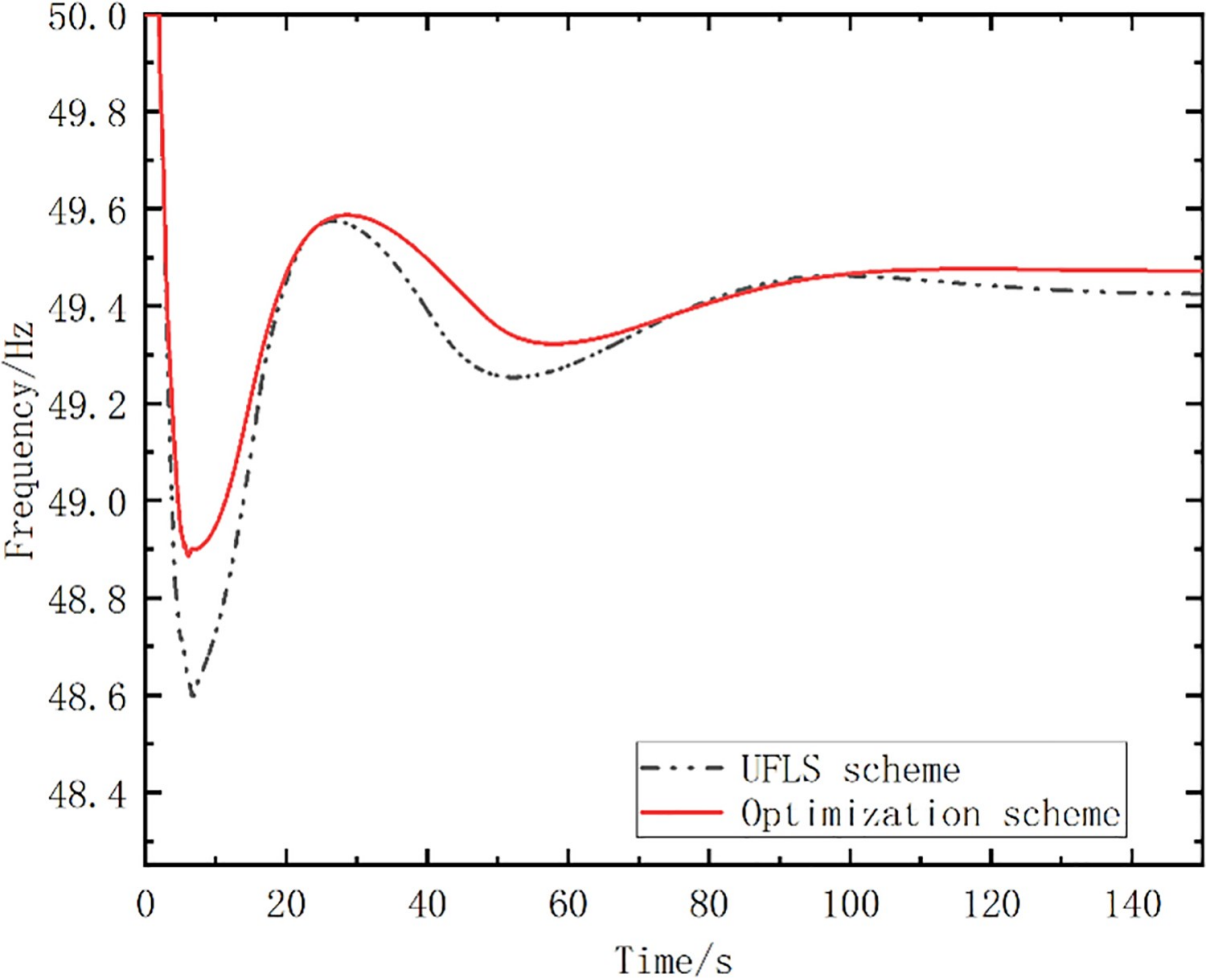
Simulated frequency curves.

Fig 12 shows the frequency curve of the system without any frequency control measures. The above parameters of the system were introduced into the proposed optimal model, and the model was solved using the proposed strategy and algorithm. One optimized scheme was obtained that included a group of optimal action frequency values and the number of action stages. The optimized scheme proposed in this paper was compared with the traditional UFLS scheme, and the load shedding setting of the UFLS was consistent with that in Table 7. The action frequency setting values of the two schemes are listed in Table 8. The simulated frequency curves are shown in Fig 13, while Table 9 lists the simulation analysis results.

**Table 8:** Two comparison schemes (Case 1).

stages	Action Frequency Setting Value (Hz)	
	Optimized Scheme	UFLS Scheme
1	49.79	49.0
2	49.73	48.8
3	49.66	48.6
4	49.58	48.4
5	49.52	48.2
6	49.00	—
7	48.94	—

**Table 9:** Simulation analysis results for two schemes (Case 1).

Schemes	*f*_min_ (Hz)	*f*_∞_ (Hz)	Δ*t_re_*(s)	*S*	Load shedding value (MW)
UFLS	48.88	49.47	103.06	52.41	1000
Optimized Scheme	48.59	49.42	133.62	93.52	2400

As shown in Fig 13 and Table 9, the lowest frequency value *f*_min_ at the first pendulum, the steady-state frequency *f*_∞_, and the time at which the frequency returns to the steady state are improved, compared with those for the traditional UFLS scheme. Although the optimized scheme has more action stages, the load shedding is far smaller than that for the UFLS scheme.

Five comparison schemes were formulated in accordance with the scheme in Table 7. Schemes 1 to 4 combined the pumped storage and UFLS for frequency recovery, but without adopting the optimization algorithm’s solution to set the action frequency. For schemes 1 to 4, the same load shedding proportion was set at the UFLS stage, and the action frequency was set to obey the frequency change principle of "first fast and then slow, first slow and then fast, and uniform change, slower in the middle and faster at both ends"

**Fig 14:**
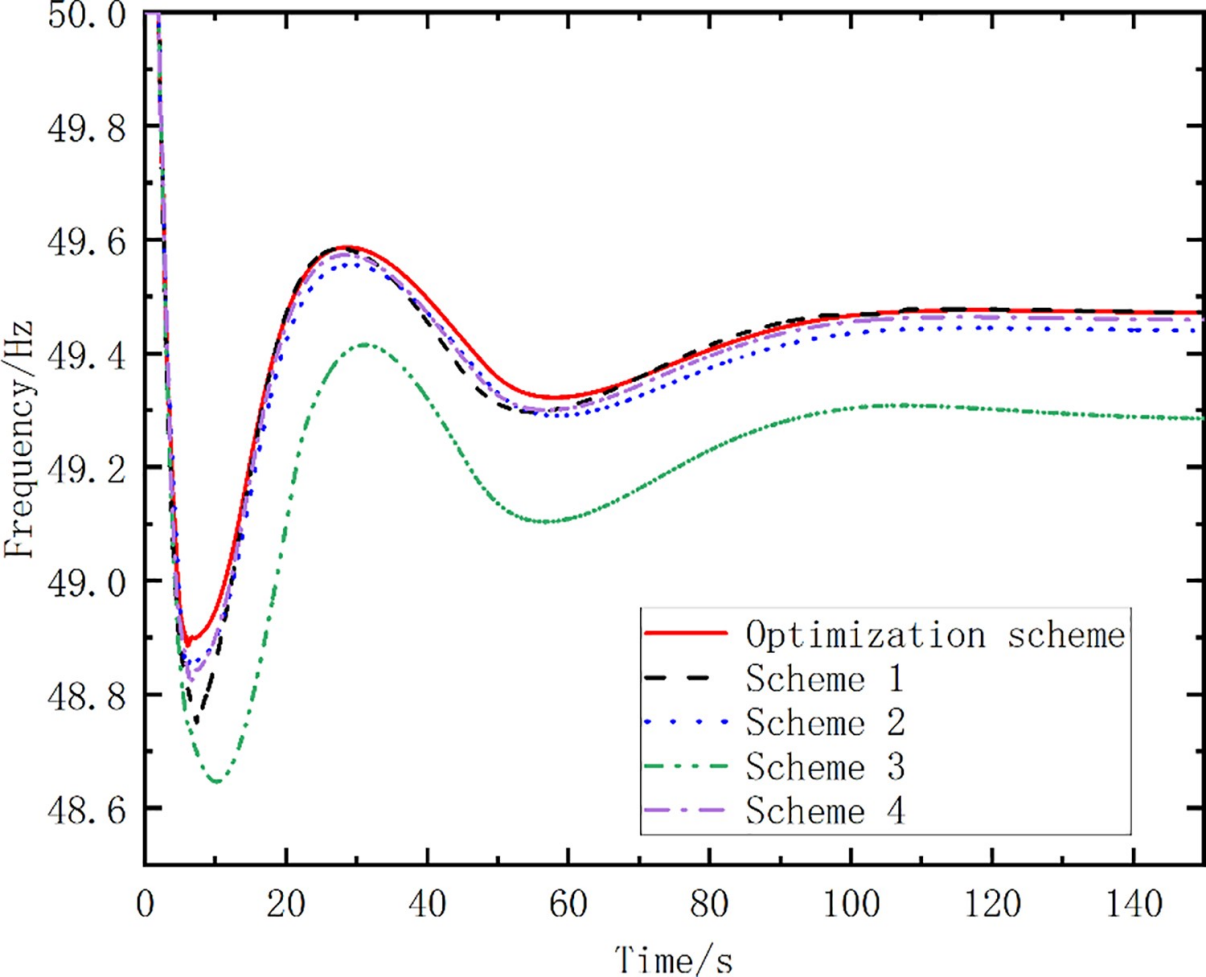
Simulated frequency curves for different schemes.

The action frequency setting values for the different schemes in Table 10 were simulated and verified by PSASP. Fig 14 shows the simulated frequency curves for the different schemes. Table 11 lists the simulation analysis results.

**Table 10:** Comparison of five schemes (Case 1).

Stages	Action Frequency Setting Value under Various Schemes (Hz)				
	Scheme 1	Scheme 2	Scheme 3	Scheme 4	Optimized Scheme
1	49.70	49.80	49.80	49.80	49.79
2	49.50	49.70	49.60	49.70	49.73
3	49.40	49.60	49.40	49.30	49.66
4	49.30	49.50	49.20	49.20	49.58
5	49.10	49.40	49.00	49.10	49.52
6	48.90	49.00	48.80	48.90	49.00
7	48.80	48.90	48.60	48.90	48.94

**Table 11:** Simulation analysis results for different schemes (Case 1).

Schemes	*f*_min_(Hz)	*f*_∞_ (Hz)	Δ*t_re_*(s)	*S*
Scheme 1	48.74	49.47	106.12	54.42
Scheme 2	48.85	49.44	104.53	59.15
Scheme 3	48.64	49.28	137.36	94.05
Scheme 4	48.82	49.45	104.25	60.73
Optimized Scheme	48.88	49.47	103.06	52.41

It can be seen from the simulation analysis results in Table 11 that the strategy proposed in this paper is superior to the other four schemes. The optimized scheme had the slowest frequency drop rate, and the amplitude of the fall was the smallest. The time Δ*t_re_* needed to recover the frequency to 49.88 Hz was 103.06 s, which was shorter than the times for the other schemes. The objective function value *S* was the smallest for the optimized scheme.

Case 2: Initial power shortage ΔP_OL0_ = 2000MW for receiving-end grid B.

**Fig 15:**
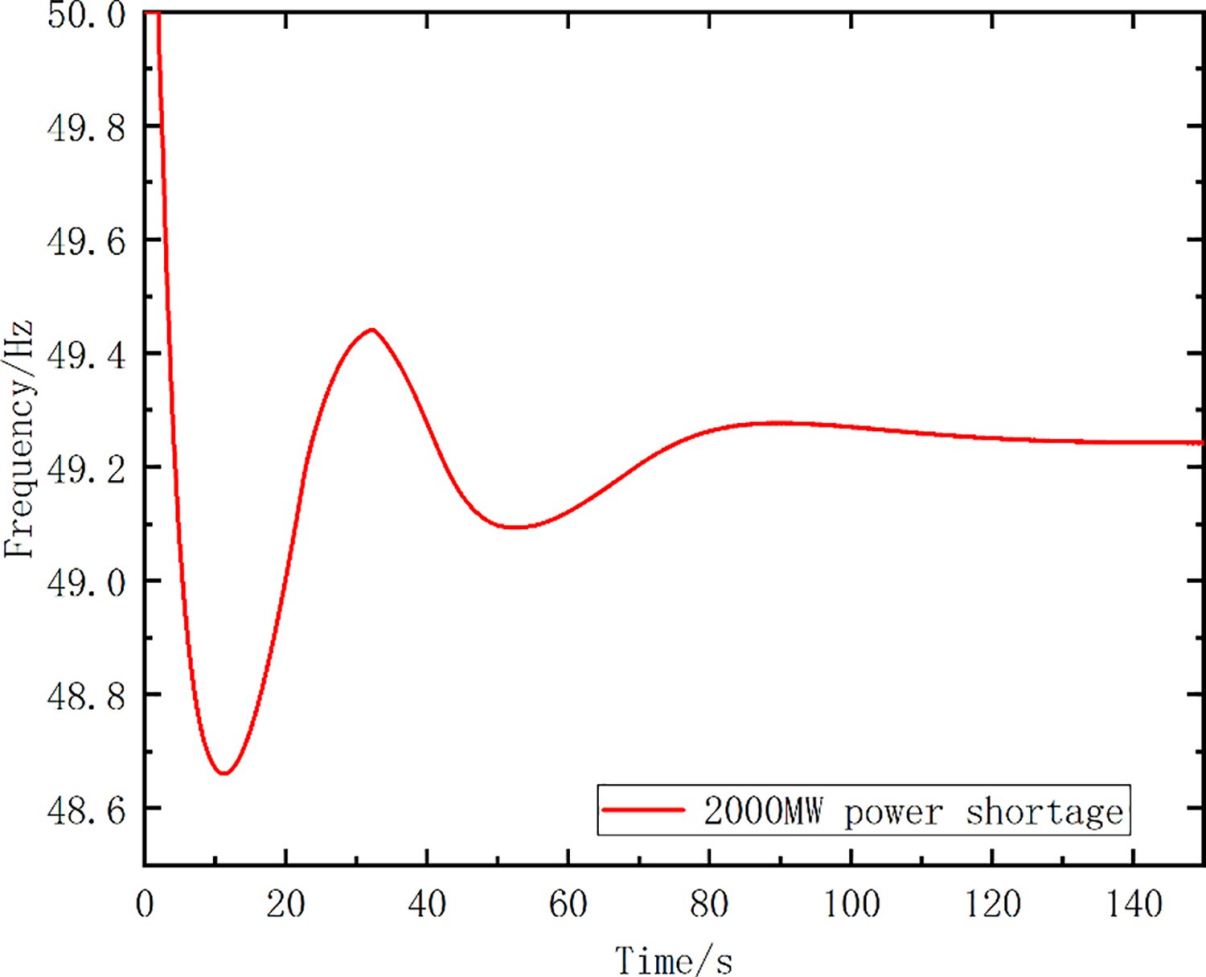
Frequency variation for the 2000 MW power shortage scenario, without any recovery strategy applied.

**Fig 16:**
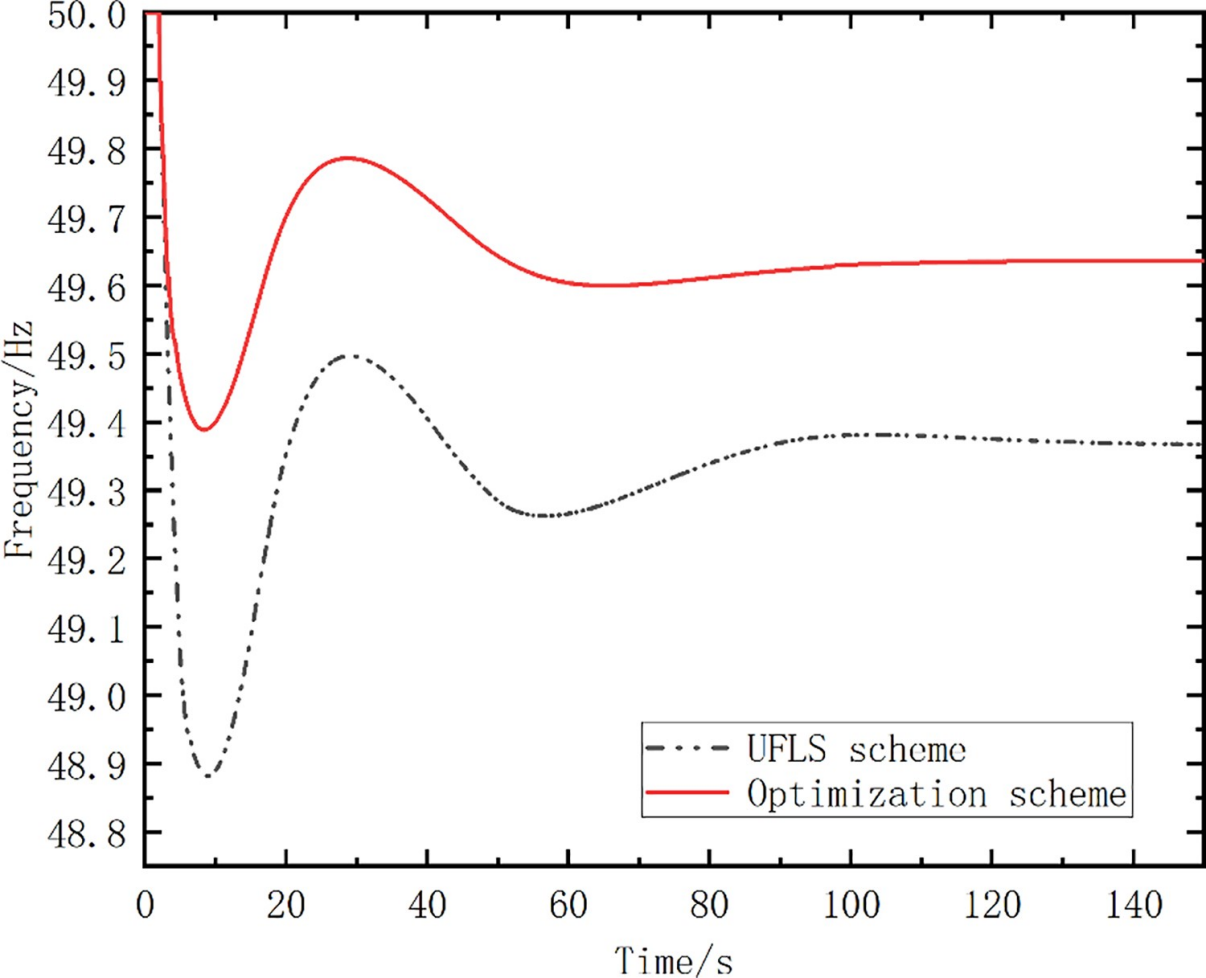
Simulated frequency curves (Case 2).

Fig 15 shows the frequency curve for the system without any frequency control strategy. The optimal action frequency and action stages for the optimized scheme were calculated using the proposed method. The optimized scheme proposed in this paper was compared with the traditional UFLS scheme, and the load shedding setting of the UFLS scheme was consistent with that in Table 7. The action frequency settings for the two schemes are listed in Table 12. The simulated frequency curves are shown as Fig 16, while Table 13 lists the simulation analysis results.

**Table 12:** Comparison of two schemes (Case 2).

stages	Action Frequency Setting Value (Hz)	
	Optimized Scheme	UFLS Scheme
1	49.78	49.0
2	49.69	48.8
3	49.64	48.6
4	49.59	48.4
5	49.43	48.2

**Table 13:** Simulation analysis results (Case 2).

Schemes	*f*_min_ (Hz)	*f*_∞_ (Hz)	Δ*t_re_*(s)	*S*	Load shedding value (MW)
UFLS	48.88	49.36	134.31	84.36	1200
Optimized Scheme	49.38	49.63	98.19	35.89	0

Table 13 shows that the optimized scheme yields better results than the UFLS scheme. Owing to the load undercutting of the UFLS scheme, the frequency cannot be restored to above 49.6 Hz, and the load-shedding amount is 1200 MW. The lowest frequency of the first pendulum frequency is 49.38 Hz, the recovery frequency is 49.63 Hz, the recovery time is 98.19 s, and the objective function value *S* is 35.89. These values are better than those for the UFLS scheme.

Table 14 compares the five schemes. The definitions of Schemes 1 to 4 here were the same as those in Case 1.

**Table 14:** Comparison of five schemes (Case 2).

Stages	Action Frequency Value under Various Schemes (Hz)				
	Scheme 1	Scheme 2	Scheme 3	Scheme 4	Optimized Scheme
1	49.80	49.70	49.80	49.80	49.78
2	49.60	49.40	49.70	49.70	49.69
3	49.40	49.30	49.50	49.60	49.64
4	49.30	49.20	49.40	49.50	49.59
5	49.10	49.10	49.30	49.40	49.43

**Fig 17:**
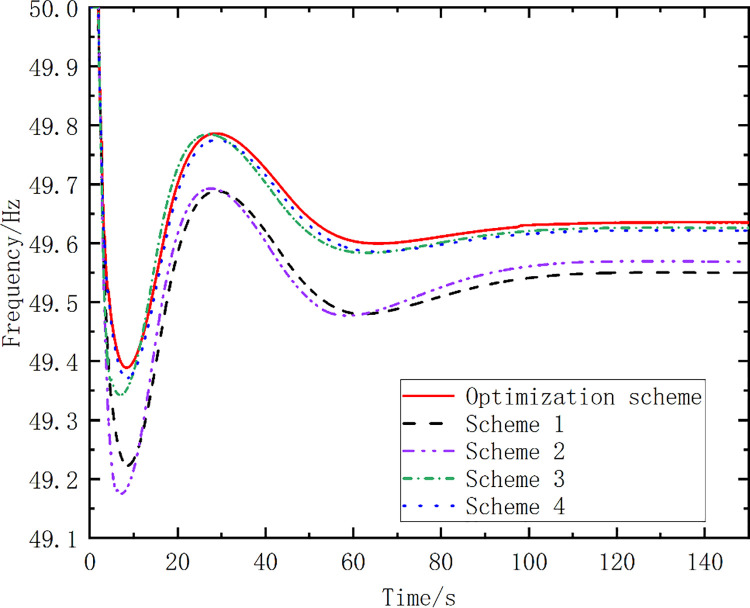
Simulated frequency curves, for the optimized scheme and the other four schemes.

Fig 17 shows the simulated frequency curves for the optimized scheme and the other four schemes. Table 15 lists the simulation analysis results.

**Table 15:** Simulation analysis results for different schemes (Case 2).

Schemes	*f*_min_(Hz)	*f*_∞_ (Hz)	Δ*t_re_*(s)	*S*
Scheme 1	49.22	49.55	119.54	50.10
Scheme 2	49.17	49.56	99.03	43.25
Scheme 3	49.34	49.62	98.64	36.94
Scheme 4	49.37	49.62	110.95	42.06
Optimized Scheme	49.38	49.63	98.19	35.89

The simulated frequency curves in Fig 17 show that the optimized scheme exhibits an obvious and effective frequency recovery effect in response to the emergency fault. According to the key effect parameters in Table 15, the frequency curve after the action of the optimized scheme exhibits the lowest frequency drop value, and the frequency control effect is better than for the other schemes. Moreover, the steady-state value *f*_∞_ after the frequency recovery was the highest for the optimized scheme, the time Δ*t_re_* was the shortest, and the area *S* was the smallest. These results validate the correctness of the proposed objective function and the effectiveness of the proposed strategy for grid frequency control.

In simulated Case 2, the power shortage of the receiving-end grid B was lower than that in Case 1, standing at 2000 MW. As can be seen from Tables 8 and 12, the optimized scheme involved fewer stages of frequency adjustment than Case 1. With respect to the fault handling, the strategy proposed in this study has good adaptability.

## 7. Conclusions

This paper explored the ways to handle the scenario in which the frequency of the receiving power grid falls sharply owing to a large power shortage after a fault on the UHV background, and proposed an adaptive under-frequency optimal control strategy for power systems, which combines the pumped storage units’ mode conversion and UFLS. The following conclusions were drawn from the present study:

1. The simulation results for different power shortage scenarios show that the proposed optimized scheme is superior to the traditional scheme at the lowest frequency point of the system, the steady-state frequency and the frequency recovery time, and has good adaptability.
2. Compared with the traditional UFLS, the proposed scheme exhibits the least load loss while ensuring the corresponding system’s stability.
3. The proposed frequency control measures compensate for the defects of excessive conventional safety control quantity and independent frequency control measures, and effectively solve the problem of emergency frequency control in response to serious power shortages at large power grids.
4. The framework of a pumped storage power station as a power energy storage system participating in the system frequency control was explored.

With the development of wide-area measurement technologies, the analysis based on the measured trajectory of the power system frequency has better application prospects for the method proposed in this paper. In the future, frequency dynamic response models will be considered, using the situational awareness technology. Because traditional UFLS can easily cause power failures in one or more areas, precise load-shedding control will also be the focus of future research.

## Abbreviations

UHV: Ultra-high voltage
UFLS: Under-frequency load shedding.
*T*_s_: Inertial time constant of the equivalent system
Δ*f*: The variation of frequency
Δ*P*_OL_: The overload or power shortage of the power system
*T*_G_: Comprehensive time constant of the system-wide generator set
Δ*P*_G_: The variation of generator power
*K*_G_: Power-frequency static characteristic coefficient of the generator set
Δ*P*_D_: The variation of load
*K*_D_: The load frequency coefficient
Δ*P*_OL0_: System’s initial overload or initial power shortage
*f*_∞_: The post-fault steady-state frequency of power system
*f_N_*: Rated frequency of power system
*T_f_*: Time constant during the system frequency decline
*K*_s_: Comprehensive power-frequency regulation coefficient
*P*_mech_: Mechanical power output of hydraulic turbine.
*K*_P_,*K*_U_: Proportional coefficient.
*D*: Net head of hydraulic turbine.
*D*_0_: Steady-state initial value of the *D*
*U*: Flow rate of the water.
*P*_0_: No-load loss.
*μ_c_*: Ideal guide vane opening.
*g*: Constant of gravity acceleration.
*L*: Length of penstock.
*U*_NL_: The critical flow rate of the hydraulic turbine from stationary to rotating.
*A_t_*: Proportionality coefficient
*y*_FL_: The actual full-load opening.
*y*_NL_: The actual no-load opening of hydraulic turbine guide vanes.
*P* ^P-S^: The absorbed power from the grid of pumped storage unit from pump mode to spinning mode.
*y*_0_: Initial opening
*k_y_*: Proportional coefficient of the closing process.
$f_{m}^{P-S}$: The action frequency value of the *m*th unit in the process from pump mode to spinning mode.
$t_{m}^{P-S}$: The action time of the *m*th unit in the process from pump mode to spinning mode.
$P_{m}^{P-S}$: The active power contribution to the grid of the *m*th unit after its operation mode are converted.
Δ*P*^P−S^: Power increment of receiving end grid after *M* pumped storage units are converted.
*k_c_*: Proportional coefficient of the opening process.
$f_{n}^{S-G}$: The action frequency value of the *n*th unit in the process from spinning mode to generator mode.
$t_{n}^{S-G}$: The action time of the *n*th unit in the process from spinning mode to generator mode.
$P_{n}^{S-G}$: The active power contribution to the grid of the *n*th unit after its operation mode are converted.
Δ*P*^S−G^: Power increment of receiving end grid after *N* pumped storage units are converted.
*H*: Number of stages of UFLS.
$P_{h}^{\mathrm{shed}}$: Load shedding value of the *h*th stage.
$f_{h}^{\mathrm{shed}}$: The action frequency value of the *h*th stage.
Δ*t*_*h*_: Time delay of the *h*th stage.
$\Delta P_{\mathrm{OL}(m)}^{P-S}$: Real-time power shortage of the system during the *m*th stage of pumped storage unit mode transition.
$f_{m}^{P-S}(t)$: Dynamic frequency trajectory in the *m*th stage of mode transition process.
$\Delta P_{\mathrm{OL}(n)}^{S-G}$: Real-time power shortage during the *n*th stage of pumped storage unit’s mode transition.
$f_{n}^{S-G}(t)$: Dynamic frequency trajectory in the process of spinning mode converts to generator mode.
$\Delta P_{\mathrm{OL}(h)}^{\mathrm{shed}}$: Real-time power shortage of the power system during the *h*th stage of the UFLS.
$f_{h}^{\mathrm{shed}}(t)$: Dynamic frequency trajectory of the *h*th stage of under frequency load shedding.
*S*^P−S^,*S*^S−G^,*S*^shed^: The area enclosed by the dynamic frequency trajectory and the rated frequency *f* =*f*_*N*_, respectively.
$f_{\min}^{P-S},f_{\max}^{P-S}$: The transient frequency safety limits of the action frequency of pumped storage units from pump mode to spinning mode.
$f_{\min}^{S-G},f_{\max}^{S-G}$: The transient frequency safety limits of the action frequency of pumped storage units from spinning mode to generator mode.
$f_{\min}^{\mathrm{shed}},f_{\max}^{\mathrm{shed}}$: The transient frequency safety limits of UFLS.
*A*: The trajectory sensitivity matrix
***η**_f_*: Transient frequency safety margin vector
η*_fi_*: Transient frequency deviation safety margin of the *i*th stage
Δ***f***_V_: Frequency variation vector
Δ*f*_*i*+1_: Frequency variation of the *i*+1th stage
***ε**_f_*: Transient frequency safety threshold value
*a_fi_*: the trajectory sensitivities of the *η*_*fi*_
*η*_*fi*_(Δ*P*_*i*_): Transient frequency deviation safety margin when the power shortage is Δ*P*_*i*_
*τ_i_*: The power increment of the power shortage Δ*P*_*i*_
*f_i_*: The action frequency of the *i*th stage
*f_cr,i_*: The frequency deviation threshold of the *f*_*i*_
*t_cr,i_*: The maximum time that the action frequency *f*_*i*_ exceeding threshold *f*_*cr*,*i*_
*t_i_*: The action starting time of the *i*th stage
*T*_0_: Initial temperature
*K*: Cooling factor
*prob*: The probability that the new solution value is accepted
Δ*E*: The variation of system energy
